# Thermal and Perceptual Responses of Older Adults With Fan Use in Heat Extremes: A Secondary Analysis of a Randomized Clinical Trial

**DOI:** 10.1001/jamanetworkopen.2025.23810

**Published:** 2025-07-29

**Authors:** Georgia K. Chaseling, Nicole T. Vargas, Lily Hospers, Hadiatou Barry, Amy Harwood, Connor Graham, Audrey-Ann Bartlett, Amélie Debray, Grant Lynch, Anthony Capon, Craig G. Crandall, Maria Fiatarone Singh, Yorgi Mavros, Peng Bi, Anil Nigam, Malorie Chabot-Blanchet, Daniel Gagnon, Ollie Jay

**Affiliations:** 1 Montreal Heart Institute, Montreal, Quebec, Canada; 2 École de Kinésiologie et Des Sciences de L'activité Physique, Faculty of Medicine, Université de Montréal, Quebec, Canada; 3 Heat and Health Research Centre, Faculty of Medicine and Health, University of Sydney, NSW, Australia; 4 Department of Sport and Exercise Sciences, Institute of Sport, Manchester Metropolitan University, Manchester, United Kingdom; 5 Monash Sustainable Development Institute, Monash University, Melbourne, Australia; 6 Institute for Exercise and Environmental Medicine, Texas Health Presbyterian Hospital Dallas, and University of Texas Southwestern Medical Center; 7 Discipline of Exercise and Sport Science, Faculty of Medicine and Health, University of Sydney, New South Wales, Australia; 8 Faculty of Health and Medical Sciences, University of Adelaide, SA, Australia; 9 Montreal Health Innovations Coordinating Center, Montreal, Quebec, Canada

## Abstract

This secondary analysis of a crossover trial examines how fan use affects body temperature, sweating, and thermal perceptions among older adults in extreme heat.

## Introduction

The US Centers for Disease Control and Prevention (CDC) advise against fan use above 32 °C (ie, 90 °F) because it can increase body temperature.^1^ Modeling suggests fans reduce body temperature up to 37 to 39 °C (ie, 99 to 102 °F), depending upon humidity.^2^ In humid heat, fan use reduced body temperature in young adults at 42 °C (ie, 108 °F),^3^ or healthy older adults at 36 °C (ie, 97 °F).^4^ Above 36 °C, fan use effects on older adults remain unclear but is likely detrimental at 42 °C or hotter.^5^ We reported that fan use with and without skin wetting reduces cardiac strain of older adults in hot and humid heat but tripled cardiac strain in very hot and dry heat.^6^ Here, we report the results for body temperature, sweating, and thermal perceptions.

## Methods

This secondary analysis of a crossover trial was approved by the University of Sydney and Montreal Heart Institute ethics committees (eMethods in Supplement 1). Reporting follows CONSORT reporting guideline, and written consent was provided (eMethods in Supplement 1). The trial protocol can be found in Supplement 2. Participants without or with coronary artery disease (CAD) underwent 3-hour exposures to hot and humid (38 °C [ie, 100 °F]; 60% humidity) or very hot and dry (45 °C [ie, 113 °F]; 15% humidity) heat. In hot and humid heat, all participants underwent 4 exposures separated by 72 hours or more, in randomized sequence: control, fan use, skin wetting, and fan use with skin wetting. In very hot and dry heat, participants with CAD only underwent control and skin wetting exposures. The prespecified secondary outcomes were the change in rectal temperature (self-inserted thermistor), sweat rate (weight change), as well as the change in thermal sensation and comfort that were measured using a 7-point and 4-point scale, respectively (ASHRAE Standard 55; reported as arbitrary units [AU]).

Outcomes were analyzed using restricted maximum likelihood-based repeated measures including a term for exposure. The covariance structure yielding the smallest Akaike information criterion, between the unstructured and compound symmetry covariance structures, was used to model within-participant errors. Statistical significance was set at *P* < .05, and results are presented with 95% CIs. SAS version 9.4 (SAS for Statistical Computing).

## Results

**Figure 1.**
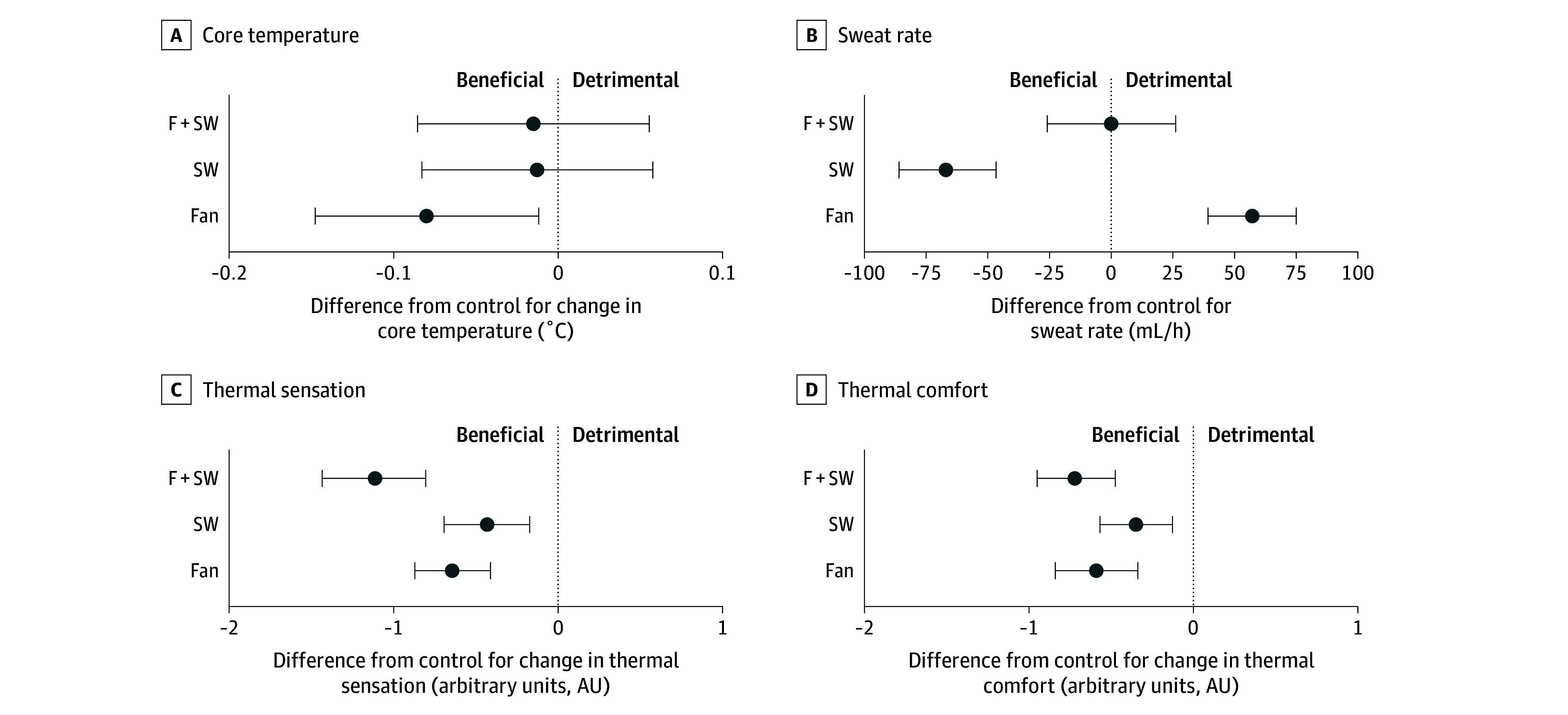
Study Outcomes During Exposure to Hot and Humid Heat (38 °C, 60% Relative Humidity) F + SW indicates exposures with combined fan use and skin wetting; Fan, exposures with fan use; SW, exposures with skin wetting.

Fifty-eight participants (31 without CAD [53%] and 27 with CAD [47%], 39 males [67%], 19 females [33%], mean [SD] age, 68 [7] years; mean [SD] height, 1.71 [0.09] m; mean [SD] weight, 79 [15] kg) underwent a total of 320 exposures that were included in the analyses (eFigure in Supplement 1). In hot and humid heat (Figure 1), rectal temperature was reduced by fan use (−0.1 [95% CI, −0.1 to 0] °C; *P* = .02), but not skin wetting alone (0 [95% CI, −0.1 to 0.1] °C; *P* = .73) or combined with fan use (0 [95% CI, −0.1 to 0.1] °C; *P* = .67). Sweating was greater with fan use (57 [95% CI, 39 to 75] mL/h; *P* < .001), reduced by skin wetting (−67 [95% CI, −86 to −47] mL/h; *P* < .001), and unaffected by fan use with skin wetting (0 mL/h [95% CI, −26 to 26] mL/h; *P* = .99). Thermal sensation and comfort improved with fan use (−0.6 [−0.9 to−0.4] AU; *P* < .001; −0.6 [95% CI, −0.8 to −0.3] AU; *P* < .001), skin wetting (−0.4 [95% CI, −0.6 to −0.1] AU; *P* = .002; −0.4 AU [95% CI, −0.6 to −0.1] AU; *P* = .002), and fan use with skin wetting (−1.1 [95% CI, −1.4 to −0.8] AU; *P* < .001; −0.7 95% CI, [−1.0 to −0.5] AU; *P* < .001).

**Figure 2.**
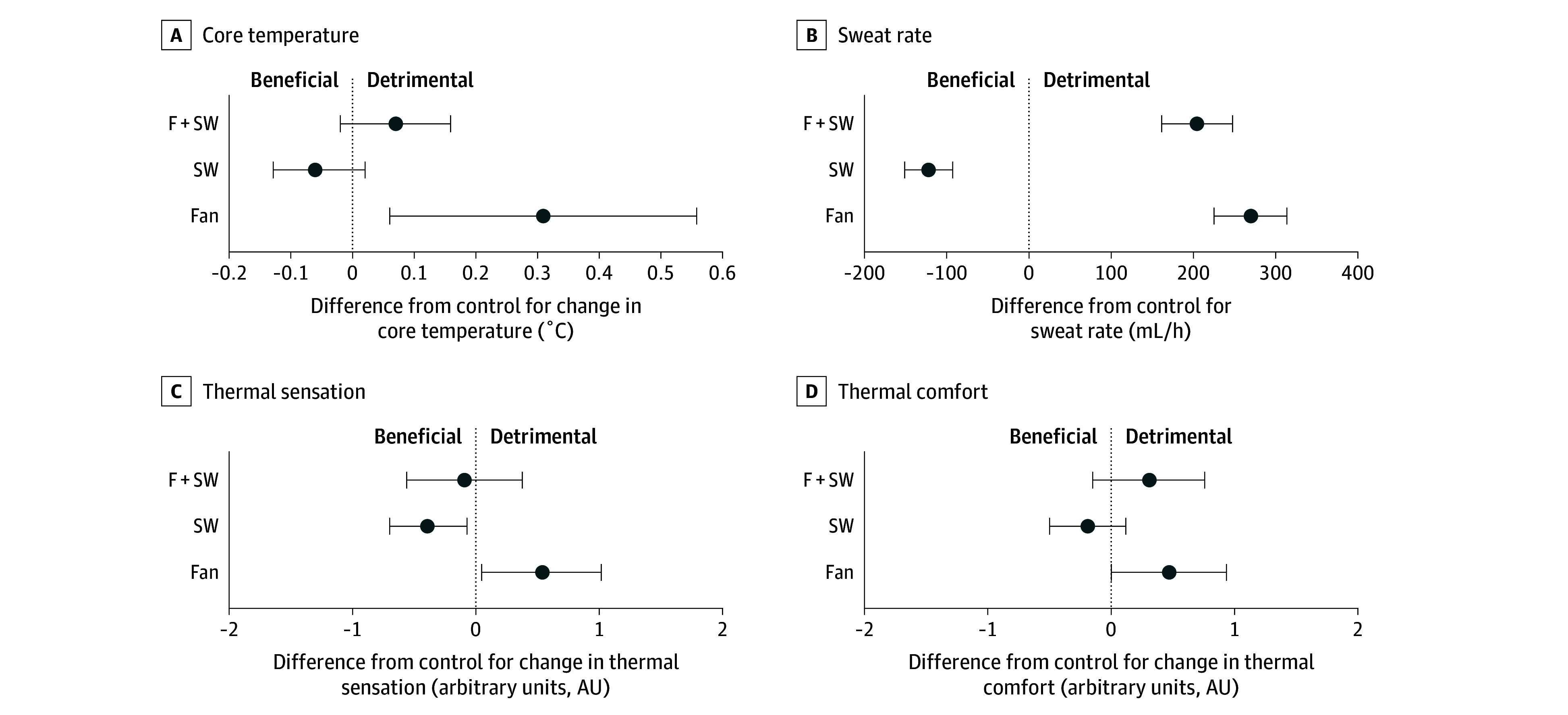
Study Outcomes During Exposure to Very Hot and Dry Heat (45 °C, 15% Relative Humidity) F+SW indicates exposures with combined fan use and skin wetting; FAN, exposures with fan use; SW, exposures with skin wetting.

In very hot and dry heat (Figure 2), rectal temperature was worsened by fan use (0.3 [95% CI, 0.1 to 0.6] °C; *P* = .02), but not skin wetting alone (−0.1 [95% CI, −0.1 to 0] °C; *P* = .13) or combined with fan use (0.1 [95% CI, 0 to 0.2] °C; *P* = .11). Sweating was greater with fan use alone (270 [95% CI, 226 to 314] mL/h; *P* < .001) and combined with skin wetting (205 [162 to 248] mL/h; *P* < .001), but reduced by skin wetting (−121 [95% CI, −150 to −92] mL/h; *P* < .001). Fan use worsened thermal sensation (0.5 [95% CI, 0.1 to 1.0] AU; *P* = .03) and discomfort (0.5 [95% CI, 0 to 0.9] AU; *P* = .047). Skin wetting improved thermal sensation (−0.4 [95% CI, −0.7 to −0.1] AU; *P* = .02), but not comfort (−0.2 [95% CI, −0.5 to 0.1] AU; *P* = .23). Fan use with skin wetting did not affect thermal sensation (−0.1 [95% CI, −0.6 to 0.4] AU; *P* = .69) and comfort (0.3 [95% CI, −0.1 to 0.8] AU; *P* = .18).

## Discussion

Contrary to CDC advice,^1^ this study found that fan use at 38 °C (ie, 100 °F), 60% humidity did not increase body temperature in older adults with and without CAD. Rather, fan use marginally decreased body temperature and improved perceptions. In very hot and dry heat, fan use worsened all outcomes and should be discouraged for these conditions. Skin wetting reduced sweating and improved perceptions in both heat conditions and could be recommended to minimize dehydration risk. Limitations include the short, laboratory-based exposures. Replication of these results in other heat-vulnerable populations should be considered.

## Notes

Supplement 1.
eMethods.
**eFigure.** Participant Flowchart Through the Study Protocol
eReferences.

Supplement 2.
Trial Protocol

Supplement 3.
Data Sharing Statement
