# Supplementary material for: Thermal and Perceptual Responses of Older Adults With Fan Use in Heat Extremes: A Secondary Analysis of a Randomized Clinical Trial
Source: JAMA Netw Open. 2025 Jul 29;8(7):e2523810. doi: 10.1001/jamanetworkopen.2025.23810 (PMC12308431; doi:10.1001/jamanetworkopen.2025.23810)
Supplement: Supplement 1. — eMethods. eFigure. Participant Flowchart Through the Study Protocol eReferences. [file jamanetwopen-e2523810-s001.pdf]

# Supplemental Online Content

Chaseling GK, Vargas NT, Hospers L, et al. Thermal and perceptual responses of older adults with fan use in heat extremes. *JAMA Netw Open*. 2025;8(7):e2523810. doi:10.1001/jamanetworkopen.2025.23810

**eMethods.**

**eFigure.** Participant Flowchart Through the Study Protocol

**eReferences.**

This supplemental material has been provided by the authors to give readers additional information about their work.

© 2025 Chaseling GK et al. *JAMA Network Open*.

## eMethods.

This supplemental material has been provided by the authors to give readers additional information about their work.

*Study design*. We conducted randomized crossover studies at the University of Sydney (Sydney, Australia) and the Montreal Heart Institute (Montreal, Canada). The data from both studies were combined and analyzed using a statistical analysis plan that was developed prior to data analysis. At both sites, the prespecified primary outcome was cardiac strain during hot/humid heat exposure and the results have been published[1]. The pre-specified secondary outcomes were the change in core temperature, sweat rate, the change in thermal sensation, and the change in thermal comfort.

*Ethical approval*. The studies were approved by the research ethics committees at the University of Sydney (#2018-496) and Montreal Heart Institute (#2019-2425). Participants were fully informed of the risks associated with the study and the possibility to withdraw at any time before providing written informed consent. The studies were registered (Australian New Zealand Clinical Trials Registry: ACTRN12618001913268, ACTRN12619000938101 and ClinicalTrials.gov: NCT03832504).

*Participants.* At the University of Sydney, only participants living without coronary artery disease were recruited. They were eligible to participate in the study if they were 60 years of age or older and were not current smokers. Participants were excluded if they presented with uncontrolled hypertension or any other chronic health condition. Participants were recruited through advertisements in the community, word of mouth, and social media. At the Montreal Heart Institute, only participants living with coronary artery disease were recruited. They were eligible to participate in the study if they were between 50 and 80 years of age and had a documented history of coronary artery disease. Participants were excluded if their condition was unstable or if comorbidities were not controlled by medications. Participants were recruited through advertisements in the community, word of mouth, social media, and cardiac rehabilitation clinics. From January 2019 to May 2023, 512 people across both sites

© 2025 Chaseling GK et al. *JAMA Network Open*.

expressed interest in the study, of whom 120 were screened for eligibility resulting in 82 being enrolled into the study. Of these 82 participants, 24 dropped out of the study before completing any heat exposure visit leaving a sample size of 58 participants who completed a total of 302 heat exposures that were included in the analyses (Figure1).

*Protocol*. At both sites, participation in the study involved up to eight laboratory visits that consisted of a 3-hour exposure to hot/humid (38˚C [~100˚F], 60% humidity) or very hot/dry (45˚C [113˚F], 15% humidity) heat within a climate chamber. These environmental conditions were chosen because heat extremes are broadly dichotomous, with high humidity events accompanied by peak temperatures in the high-30˚C's [high-90˚F's/low-100˚F's] (e.g., Chicago 1995; 39˚C [102˚F], 50% humidity) and very hot events being typically arid (e.g., 2021 Pacific Northwest; 44-47˚C [112-117˚F], <20% humidity). For hot/humid exposures, all participants from both sites underwent four interventions in a randomized sequence: 1) no intervention (control); 2) electric fan use; 3) skin wetting; 4) fan use with skin wetting. For very hot/dry exposures, we anticipated that fan use would worsen physiological heat strain. For this reason, participants living without coronary artery disease (Sydney site) underwent the four interventions whereas participants living with coronary artery disease (Montreal site) only underwent the control and skin wetting interventions. Prior to entering the climate chamber, baseline data were collected in a 22˚C [~72˚F] room. Within the climate chamber, participants remained seated upright on a chair, and they were provided water (3 ml/kg of body mass every hour) to minimize dehydration. Participants were free to withdraw at any time and the protocol was terminated by the investigators if pre-determined criteria were attained (see Protocol). Within participant, exposures were performed at the same time of day and were separated by a wash-out of ≥72 hours. Participants were asked to avoid strenuous exercise (24 hours), caffeine (12 hours), and alcohol (12 hours) and to consume 250 ml of water two hours prior to each exposure.

© 2025 Chaseling GK et al. *JAMA Network Open*.

Participants living with coronary artery disease who had a prescription for calcium channel blockers were also asked to withhold this medication for 48 hours prior to each exposure.

*Measurements.* Body temperature was measured as rectal temperature (TM400, Covidien, Mansfield, MA, USA). Sweat rate was calculated from changes in nude body weight, accounting for fluid intake and urine output, and divided by heat exposure duration. Thermal perceptions were measured as thermal sensation and thermal comfort using a 7-point and 4-point scale, respectively (ASHRAE Standard 55).

*Trial outcomes.* The pre-specified secondary outcomes were the change in rectal temperature (average of last 5 minutes of heat exposure minus the last 5 minutes of baseline), sweat rate calculated from changes in nude body weight, and the change in thermal sensation and thermal comfort (last measurement during heat exposure minus measurement at baseline).

*Statistical analyses.* As per the statistical analysis plan, the outcomes were analyzed with linear mixed models that included a term for strategy and that accounted for repeated measures. Contrasts under this model allowed for three comparisons: 1) control vs. fan use; 2) control vs. skin wetting; 3) control vs. fan use with skin wetting. These contrasts were tested at the 0.05 significance level and are presented with point estimates along with 95% confidence intervals. Statistical analyses were performed using SAS Version 9.4.

© 2025 Chaseling GK et al. *JAMA Network Open*.

**eFigure.** Participant Flowchart Through the Study Protocol

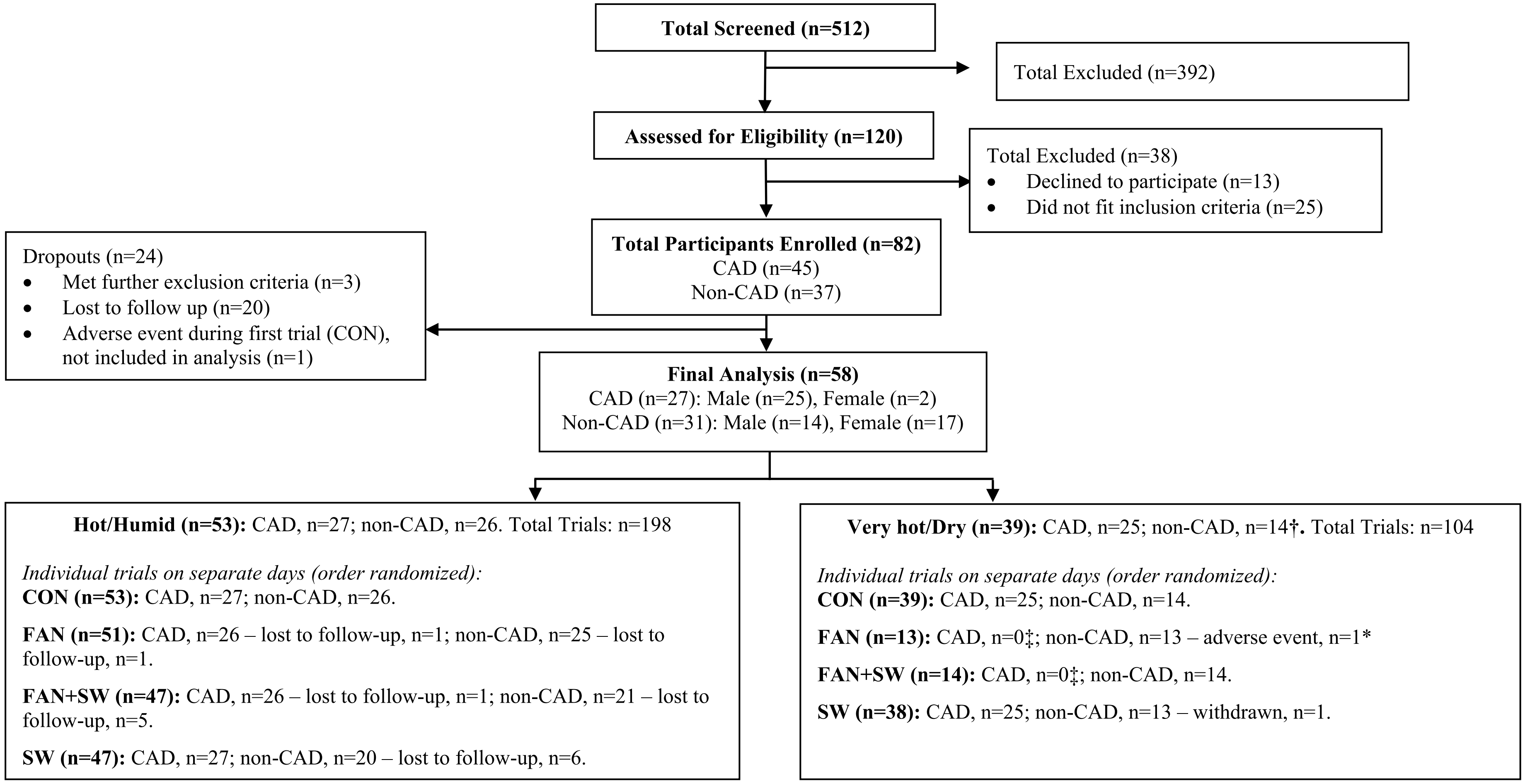


© 2025 Chaseling GK et al. *JAMA Network Open*.

**eReferences.**

1. Chaseling GK, Vargas NT, Hospers L, et al. Simple Strategies to Reduce Cardiac Strain in Older Adults in Extreme Heat. *N Engl J Med*. Nov 7 2024;391(18):1754-1756. doi:10.1056/NEJMc2407812

© 2025 Chaseling GK et al. *JAMA Network Open*.

*†Very hot/Dry (VHD) trials in non-CAD participants halted after 14 participants underwent the FAN trial in VHD conditions: 6 (5 females) of 14 (6 females) participants (43% of all participants; 83% of female participants) could not complete 180-min exposure with FAN use. In contrast, all non-CAD participants who undertook CON, FAN+SW, and SW trials completed 180 minutes of exposure in VHD conditions (total trials, n=41). *Adverse event at pre-exposure baseline (non-sustained ventricular tachycardia) for 1 participant prior to starting FAN trial, which was the 3rd exposure in VHD conditions – participant withdrew but data from previous 2 trials (CON, FAN+SW) are included in the analyses. ‡ FAN and FAN+SW trials in VHD conditions not conducted for CAD participants due to safety concerns.*

© 2025 Chaseling GK et al. *JAMA Network Open*.
