# Supplementary material for: Thermal and Perceptual Responses of Older Adults With Fan Use in Heat Extremes: A Secondary Analysis of a Randomized Clinical Trial
Source: JAMA Netw Open. 2025 Jul 29;8(7):e2523810. doi: 10.1001/jamanetworkopen.2025.23810 (PMC12308431; doi:10.1001/jamanetworkopen.2025.23810)
Supplement: Supplement 2. — Trial Protocol [file jamanetwopen-e2523810-s002.pdf]

Original protocol – USyd #2018-496: Use of fans and/or skin-wetting as a low cost cooling strategy for older adults during heatwaves

Funding organizations
National Health and Medical Research Council

Principal investigator
Ollie Jay (PhD)

Collaborators
Yorgi Mavros, PhD
Maria Fiatarone Singh, PhD
Lily Hospers

Date of initial protocol approval (Version 01)	29-08-2018

1- Outline the theoretical, empirical and/or conceptual basis, background evidence for the research proposal with relevant literature.

The frequency, intensity and duration of heat waves are increasing globally (Bi et al. 2011), and with these increases we can expect with a high level of certainty a parallel rise in heat-related morbidity and mortality (Hajat, O'Connor & Kosatsky, 2010). In an attempt to combat this, there is a need for evidence-based guidelines to be made available that educate people on the steps they can take to protect themselves during extreme heat events. One particular population group at a disproportionately higher risk of morbidity and mortality during these extreme weather events is the elderly (Hajat, O'Connor & Kosatsky, 2010). Therefore, it is of great importance to make targeted recommendations for this population.Air-conditioning is the best protection during extreme heat events, but it is not accessible to many of the most vulnerable populations and widespread use and reliance on it during extreme heat events can cause blackouts (Kaiser et al., 2001; Semenza et al., 1996). Researchers began examining the effectiveness of using a fan as a cooling intervention and found that public health recommendations regarding fan-use were not uniform between agencies and often not evidence-based (Jay et al., 2015). If fan-use were to be found effective, it would provide a low-cost solution with a low-energy requirement. Research to date suggests that the benefits of fans have been greatly underestimated by public health agencies (Jay et al., 2015). Despite organizations such as the WHO, EPA and the Government of Victoria advising against using fans in hot conditions, a study published in the Journal of the American Medical Association (JAMA) demonstrated that fan use reduced physiological strain in young healthy men in simulated hot/humid heat wave conditions (Ravanelli et al., 2015). The use of a fan increased airflow which improved sweating efficiency and led to greater evaporative heat loss. The effectiveness of fan-use does depend on both the unique environmental conditions and the individual; it has been suggested that in certain conditions fans may be less effective and even harmful to older adults due to age-related decrements in sweating (Gagnon et al., 2016). Due to the findings to date it is clear that there is a need for evidence based recommendations specific to both the environmental conditions and population-specific, particularly those populations who have compromised thermoregulatory ability, such as the elderly. In a recent article by Jay and Capon (2017) it was proposed that external skin wetting could be used by the elderly to compensate for their decreased sweating ability, while still gaining the benefits of increased latent heat loss as a result of improved evaporative efficiency with fan-use. This combination has the potential to be a simple, cost-effective cooling intervention that has 50-times lower cost and electricity requirement than air conditioning and so therefore can be utilized by vulnerable populations in a low-resource environment. We propose the below study to examine if fan-use, in combination with skin-wetting can be an effective cooling strategy that is able to decrease the physiological strain experienced by elderly during heat waves, and as a result decrease their chance of experiencing an adverse cardiovascular event. During the 2003 heat wave in Lyon, France, antihypertensive use was associated with a 177% increased risk of mortality in adults presenting to the emergency department with heat stroke (Argaud et al., 2007). Due to this historical evidence and the prevalence of hypertension in older adults, it is important to include older adults on antihypertensives in our study to adequately represent the older adult population, while also investigating the impact of antihypertensive use on individuals' responses to simulated heat wave exposure and the proposed interventions. We believe the proposed project will provide evidence

that will help public health and government organizations make evidence-based environment and population specific recommendations for older adults during heatwaves.

2- Outline the methodology for the research proposal including:

2.1. Research Questions:

The primary research question guiding this project is: Are fans and/or skin wetting effective in decreasing the physiological strain experienced by elderly during a simulated hot humid heatwave (38°C with 60% relative humidity).

The secondary research question guiding this project is: are fans and/or skin-wetting effective in decreasing the physiological strain experienced by elderly during a simulated hot, dry heatwave (46°C with 10% relative humidity).

2.2 Research hypotheses:

The research hypotheses for this study are:

1. In the hot and humid condition, all interventions will result in improved cooling compared to the control (no-fan) trail. With fan and skin-wetting (FSW) being the most effective, then no fan and skin-wetting (NFSW), then fan (F), and finally, the control, no-fan (NF).
2. In the very hot and dry condition, skin wetting with no fan (SWNF) will be the most effective cooling intervention followed by fan and skin-wetting (FSW). We expect the fan (F) intervention to be less effective than the control, no-fan (NF).

2.3 *Participant characteristics:* To complete this study, 56 (greater than or equal to 60 years) adults will be recruited from previous participants who have consented to being contacted for future studies and through the distribution of recruitment flyers at both the Cumberland and Camperdown campuses at The University of Sydney.

2.4 *Sample size:* The sample size computation is based on comparison of the primary endpoint (change in rate pressure product from baseline to the end of a 2 h simulated heatwave exposure to 38ºC and 60% relative humidity) between the 4 conditions of control, fan use alone, skin wetting alone, fan use + skin wetting. This preliminary data from young healthy participants was provided by Nate Morris, a former PhD student from within our laboratory. A non-repeated measures ANOVA on this data demonstrates 20% (eta squared of 0.2) of the variability was due to the intervention. Using GPower 3.1.9.2 software, an ANCOVA: Fixed effects, main effects and interactions f-test was performed using an alpha of 0.05, beta of 0.1 and an effect size of 0.5 to determine a total sample size of 45 is needed to obtain sufficient statistical power. To account for approximately 20% loss to follow up, 56 participants will be included in the study. Of the 56 participants, 14 will be healthy older females, 14 will be healthy older males, 14 will be healthy females taking antihypertensive medication, and 14 will be older males taking antihypertensive medication.

2.5 *Inclusion/Exclusion Criteria:*

Inclusion Criteria: Non-smokers or ex-smokers (greater that or equal to 1y since quitting), fluent/have no difficulty understanding and speaking English, and age of greater than or equal

to 60 y. Age will be matched across all groups. Participants with hypertension must have been diagnosed as hypertensive and taking antihypertensive medications.

Exclusion criteria: body mass index greater than or equal to 35. No history/signs/symptoms of coronary artery disease or heart failure. Currently undertaking hormone replacement therapy. Currently taking medications known to cause hyper- or hypo-hidrosis (except those taken for hypertension). Currently taking a beta-blocker. Evidene of current fluid and electrolyte disorders, anemia, abnormal thyroid function, arrhythmias, diabestes, renal disease, liver disease, cerebrovascular disease, significant pulmonary disease, endocrine abnormalities, uncontrolled hypertension (180/110mmHg), significant cognitive impairment, psychiatric disorder, substance abuse, degenerative neurological condition or any other medical condition deemed to pose risk during the proposed testing or experiments, or preclude them from completing the screening stress test.

2.6 *Data collection protocol:*

The 2 preliminary sessions and the 4 experimental sessions (with the option of doing an additional 4 experiemental trials) will take place at the University of Sydney, Cumberland Campus. The time involvement will be approximately 2 hours for the first preliminary session, 1 hour for the second preliminary session and 3 hours for each of the experimental sessions. This involves a total time commitment of approximately 15 hours (or approximately 27 hours if the participant chooses to do an additional four trials).We are recruiting participants for at least four experimental sessions that will take place in the hot, humid condition and providing them with the option of doing an additional four experimental sessions that will be carried out under a different simulated heatwave condition (very hot, dry). These two simulated heatwaves provide very different interactions between the individual, the environment and the proposed interventions and so it is of great importance to study both types. For example, in a previous study carried out in our lab a fan was beneficial in hot-humid conditions due to the increased airflow increasing sweating efficiency and therefore evaporative heat loss, whereas in the hot-dry condition the increased airflow accelerated dry heat gain and so fan-use was actually unfavourable. We understand that the two preliminary sessions and 8 experimental sessions are a big time commitment and so we have chosen to take the above approach to minimize attrition for the primary objective. Investigating the low cost at-home cooling interventions in the hot, humid condition is our primary objective as these are the types of heatwaves most common worldwide. Investigating the same interventions in the very hot, dry condition allows us to investigate their impact in the type of heatwave most prevalent in Australia.

Pre-screening and preliminary sessions: Participants will be initially screened over the phone using a standardized phone screen based on the inclusion and exclusion criteria of our study. If the participants pass the phone screen they will be invited in for a preliminary session. The first preliminary session involves a complete medical history, physical examination and cardiac stress test. This screening will be carried out by geriatrician Maria Fiatarone Singh, MD. During the second preliminary session the participant will be asked to arrive fasted and their body composition will be determined and a full blood count will be taken. These preliminary sessions are important to further determine their eligibility for the study and deem them safe to participate. The following equipment and techniques will be used:

•Body Composition: Body composition will be determined using dual x-ray absorptiometry, which is an enhanced form of x-ray technology that will assess the distribution of muscle, fat, water and bone within their body.

•Cardiac Stress Test: The participant will undergo a physician-supervised graded exercise test on a treadmill while undergoing ECG and blood pressure monitoring. Both are described below.

•Venepuncture: A blood sample will obtained by peripheral puncture of the forearm vein during their second preliminary session. Venepuncture will be performed by personnel with phlebotomy training.

If the participant agrees to participate in this study, they will undergo testing at the Cumberland Campus of the University of Sydney for approximately three hours on 4 separate occasions. (There is an option to participate in an additional four experimental sessions.) Each experimental session will be separated by at least 72 hours. Participants will be asked to abstain from alcohol and caffeine, avoid strenuous exercise in the 12 h prior to each experimental session, and will be instructed to consume a light meal and 0.5L of water ~2 h before arriving at the lab. They will be provided with a standardized singlet and shorts to wear and will undergo 30-min of baseline rest (seated) in a thermoneutral room (~24°C), where they will be instrumented using the equipment described below. After 30-min, they will then enter a climate-controlled chamber, where they will be seated unrestrained in a standard armchair for the remainder of the 2-hour experimental protocol. This protocol will consist of the participant sitting in a 38ºC and 60% relative humidity room. (If they choose to participate in the additional four trials the conditions will be 46ºC and 10% relative humidity). During each session the participant will undergo one of the following four conditions: no cooling intervention, use of an electric fan, self-wetting the skin with a sponge, or use of an electric fan while self-wetting the skin with a sponge. Additionally, in all trials they will be asked to drink 250 ml of cold (~18ºC) water every half-hour (750 ml total). In all fan trials, they will be seated 1.0 m from an 18” diameter fan set to the highest setting. The following equipment and techniques will be used:

•Rectal temperature sensor: The participant will be asked to insert a flexible sensor 10-12cm into their rectum. A marker is placed on the sensor using sterile surgical tape. The participant will insert the sensor until the tape reaches their anal surface. The insertion of the sensor may cause some mild discomfort and minor irritation; however, this sensation soon passes. The participant will receive proper instruction regarding the placement of the sensor to ensure their safety and comfort. The participant will be responsible for the insertion of this sensor. It will provide the researcher with an indication of the amount of heat stored in their body and will be tracked throughout the entirety of each experimental session

•Skin temperature sensors: Eight skin sensors will be taped to the participants skin surface with hypoallergenic tape. Some hair may need to be shaved (by the use of disposable razors) in order to secure the probes adequately. These probes give an indication of skin temperature and heat loss from the skin and will be recorded throughout the entirety of each experimental session.

•Blood pressure: An automated blood pressure monitor will be strapped to the participants arm and blood pressure will be taken every fifteen minutes during the experimental protocol and will be used by researchers to better characterize skin and limb blood flow and calculate RPP

(heart rate x systolic blood pressure). Blood pressure will also be taken three times during the orthostatic intolerance test described below.

•ECG monitoring: 12 soft electrodes will be stuck to the participants torso and will measure the electrical signals of their heart. This will produce signals on a display that will be continuously monitored to ensure the participants safety throughout the cardiac stress test during the first preliminary session and each experimental session.

•Heart rate: Heart rate will be measured using the ECG monitoring.

•Skin blood flow: A flexible laser probe will measure skin blood flow non-invasively at the upper back during the entirety of each experimental session. This measurement device does not result in any discomfort or residual medical effects.

•Limb blood flow: Limb blood flow will be determined using venous occlusion plethysmography. This technique involves a rapidly inflating cuff around the participants arm to a pressure of ~60 mmHg (compared to ~180 mmHg during a blood pressure reading). A strain gauge that resembles an elastic band will be placed on their forearm and this will record minor changes in limb volume. This measurement will be taken every thirty minutes during each experimental session.

•Ventilated sweat capsules: A small plastic capsule connected to plastic tubing will be placed on the participants upper back and forearm. Dry air is passed through this capsule and a humidity sensor will pick up humidity from the skin and provides a measurement of local sweat rate throughout each experimental session.

•Cognitive test: The participant will be asked to complete a short-form of the Stroop Colour and Word Test (SCWT) immediately before and at the completion of the heatwave exposure. This test involves them looking at various words and identifying which color the words are shown in.

•Whole-body sweat loss: The participant will be weighed on a platform scale immediately before and at the completion of the heatwave exposure in order to compare whole-body sweat losses.

•Rating of thermal comfort and sensation: The participant will be asked to rate how warm they feel and how uncomfortable the heat makes them on two separate visual analogue scales every fifteen minutes during each experimental session.

•Nausea and lightheadedness scale: The participant will be asked to rate how much (if any) nausea or lightheadedness they are experiencing and tick if they are experiencing any other symptoms (including paleness, muscle cramps, tiredness, weakness and headache) every fifteen minutes during each experimental session.

•Orthostatic hypotension: At the completion of the heatwave exposure the participants blood pressure will be taken. They will then be asked to stand up and their blood pressure will be taken after 1 minute of standing and then again after 3 minutes of standing. This test is carried

out to see if there is evidence of postural hypotension (a drop in blood pressure within 3 minutes of standing from a seated position).

All equipment and procedures described above are commonly used procedures and have been used multiple times by the investigators conducting the study (1-4, 9, 10) as well as research groups (5-8) in the field of thermoregulation across the world and these methods are all in accordance with the Declaration of Helsinki.

2.7 Statistical Analysis:

The primary research outcomes for this study are rectal temperature (Tre), rate pressure product (RPP), heart rate (HR), blood pressure (BP), thermal sensation (TS), thermal comfort (TC) and pre to post trial whole-body sweat losses (WBSL).Secondary outcomes to be compared are skin temperature (TSk), limb blood flow (LBF), skin blood flow (SkBF), local sweat rate (LSR), cognitive performance, rating of nausea and light headedness (NL), and evidence of orthostatic hypotension (OH).All data will be compared between all four interventions and both conditions (very hot and dry, and hot and humid).To assess the primary and secondary outcome variables, pre to post trial changes as well as the means of the dependent variables will be analyzed using one-way repeated measures ANOVAs with the repeated factor of cooling intervention (four levels: no fan (NF), fan (F), fan and skin-wetting (FSW), and no fan and skin-wetting (NFSW)) will be employed. If significant main effects or interactions are found, independent differences will be assessed using a two-tailed paired Student's t-tests while maintaining a fixed probability (5%) of making a type I error using a Holm-Bonferroni correction. All statistical analyses will be performed with GraphPad Prism (version 6.0, GraphPad Software, La Jolla, CA).

3- References

1. Argaud, L., Ferry, T., Le, Q. H., Marfisi, A., Ciorba, D., Achache, P., ... & Robert, D. (2007). Short-and long-term outcomes of heatstroke following the 2003 heat wave in Lyon, France. Archives of internal medicine, 167(20), 2177-218

2. Bi, P., Williams, S., Loughnan, M., Lloyd, G., Hansen, A., Kjellstrom, T., ... & Saniotis, A. (2011). The effects of extreme heat on human mortality and morbidity in Australia: implications for public health. Asia Pacific Journal of Public Health, 23(2_suppl), 27S-36S.

3. Gagnon, D., Romero, S. A., Cramer, M. N., Jay, O., & Crandall, C. G. (2016). Cardiac and thermal strain of elderly adults exposed to extreme heat and humidity with and without electric fan use. Jama, 316(9), 989-991

4. Hajat, S., OConnor, M., & Kosatsky, T. (2010). Review: Health effects of hot weather: from awareness of risk factors to effective health protection. The Lancet, 375856-863. doi:10.1016/S0140-6736(09)61711-6

5. Jay, O., & Capon, A. (2018). Use of physiological evidence for heatwave public policy. The Lancet Planetary Health, 2(1), e10.

6. Jay, O., Cramer, M. N., Ravanelli, N. M., & Hodder, S. G. (2015). Should electric fans be used during a heat wave. Applied ergonomics, 46, 137-143

7. Kaiser R, Rubin CH, Henderson AK, Wolfe MI, et al. Heat-related death and mental illness7.during the 1999 Cincinnati heat wave. Am J Foren Med Path. 2001;22(3):303-7

8. Ravanelli, N. M., Gagnon, D., Hodder, S. G., Havenith, G., & Jay, O. (2017). The biophysical and physiological basis for mitigated elevations in heart rate with electric fan use in extreme heat and humidity. International journal of biometeorology, 61(2), 313-323.

9. Ravanelli, N. M., Hodder, S. G., Havenith, G., & Jay, O. (2015). Heart rate and body temperature responses to extreme heat and humidity with and without electric fans. Jama, 313(7), 724-725.

10. Semenza JC, Rubin CH, Falter KH, Selanikio JD, Flanders WD, Howe HL, et al. Heat-related11.deaths during the July 1995 heat wave in Chicago. New Engl J Med. 1996;335(2):84-90.

Original protocol – MHI 2019-2425: Identifying optimal cooling strategies for coronary artery disease patients during heatwaves

Funding organizations
National Health and Medical Research Council

Principal investigator
Daniel Gagnon (PhD)

Collaborators
Martin Juneau (MD)
Anil Nigam (MD)
Céline Ferland (Inf.)
Julie Lalongé (R.A.)

Date of initial protocol approval (Version 01)	07-09-2018

1- Introduction and definition of research problem

Periods of extreme heat have been on the increase for several years now. In 2004, it was predicted that these climatic events would occur more frequently, be more intense and last longer[1]. Periods of extreme heat can have catastrophic consequences for human health and well-being. The most striking example is the heatwave that hit Europe in 2003, causing the death of 70,000 people, including 40,000 in the space of 2 weeks[4]. Since, many public health agencies have developped action plans to deal with periods of extreme heat, in recognition of the danger posed by such events[5-11].

Several studies have assessed the risk factors leading to hospitalisation and death during periods of extreme heat. It is now well established that the elderly are most at risk of hospitalisation and death during such events[12-14]. However, it is important to consider that hospitalisations and deaths of elderly people during periods of extreme heat are not directly caused by excessive body hyperthermia (e.g., heatstroke). Rather, they are primarily driven by adverse cardiovascular events[15]. These observations can be explained by the cardiovascular demands imposed by heat exposure. During heat exposure, vasodilation of cutaneous blood vessels promotes heat exchange between the body and the environment. However, this vasodilation results in a fall of peripheral resistance, which must be compensated for by an increase in cardiac output to maintain blood pressure. The cardiovascular demands imposed by heat exposure could exceed the ischaemic threshold of people with known or silent coronary artery disease, predisposing them to a risk of adverse cardiovascular events even if body temperature does not reach a level associated with, for example, heatstroke.

Several studies have sought to better understand the effects of human ageing on heat loss responses (sweat production, cutaneous vasodilation), as well as on cardiovascular responses during heat exposure. Relatively fewer studies have attempted to identify simple and effective interventions to alleviate cardiovascular strain of older adults during extreme heat exposure. We recently published a pilot study that evaluated the impact of fan use on thermal (core temperature) and cardiovascular (heart rate) strain of healthy older adults (60-80 years of age)[2]. A previous study showed that the fan use reduced thermal and cardiovascular strain of healthy young adults (20-30 years of age) during exposure to a 36°C and 42°C environment[3]. In contrast to these observations, we found that fan use resulted in greater thermal and cardiovascular strain in healthy older adults during a exposure to a 42°C environment. We believe that this observation is explained by the fact that fan use does not result in a net improvement in heat loss under these conditions, due to the limited capacity of older adults to produce sweat[16,17]. Such an observation raises the need to identify alternative solutions that could be recommended to alleviate cardiovascular strain of older adults during heatwaves. It would be especially important to identify such interventions for people with cardiovascular disease, since this population is at an even greater risk of hospitalisation and mortality during periods of extreme heat. The aim of this study is to evaluate simple and ecological interventions to alleviate cardiovascular strain of adults with coronary artery disease.

2- Relevant literature review

The devastating health impacts of extreme heat events are clear across the globe. For example, a heatwave caused 70 000+ excess deaths in Europe during the summer of 2003. Mortality/morbidity rates during heatwaves are especially elevated in people with coronary artery disease (CAD)[18]. A lower sweating capacity that occurs with ageing renders CAD patients more vulnerable to

overheating, potentially predisposing them to ischemic events in extreme heat[14,19]. Risk may be further compounded in CAD patients prescribed cardio-selective beta-blockers, due to a blunted skin blood flow that may further compromise heat dissipation[20,21].

By far the most effective cooling strategy during a heatwave is air conditioning (AC) use[14]. However, access to AC is not universal and economical concerns limit its use by those who do have access[22]. Reliance upon AC also places significant burden on the electrical grid, which can cause brown-outs or black-outs during periods of extreme heat, and it adds a substantial thermal load to the environment [23,24]. In contrast, electric fans offer a cooling strategy with a 50-fold lower power requirement (55-100 W vs. 1500-5000 W) and cost ($64/year vs. $850-$2950/year) compared to AC. The energy benefits of this approach are two-fold: the individual is targeted instead of the surrounding space, and no active cooling of the air is required. During the 1990s, fan distribution programs targeted the most vulnerable in cities such as New York and Chicago as part of their heat wave management strategy[25]. But these programs were discontinued due to concerns that fans paradoxically increase the risk of heat-related illnesses. In fact, all major public health agencies (e.g. WHO, CDC) now insist that fans should be turned off when ambient temperature exceeds ~35°C[5-9] because they can supposedly speed the onset of heat exhaustion and critically exacerbate dehydration. It should be noted that, despite such recommendations, a 2012 Cochrane review concluded there is no evidence to support or refute the use of electric fans during heatwaves[26].

The notion that fan use may be detrimental in extremely hot conditions mostly likely arises from the understanding that as air temperature exceeds skin temperature (at ~35°C), more heat will flow into the body with additional air movement. After all, this is the basis upon which fan-assisted ovens accelerate cooking time. However, unlike a turkey in an oven, humans secrete sweat on to the skin surface when heated and the subsequent evaporative heat loss serves to greatly cool the body. Moreover, this evaporation is greatly enhanced with increased air velocity; leading to the evaporation of sweat that would otherwise sit on the skin, or drip off the body and provide no cooling. Therefore, it becomes clear that only when the additional heat gain with a fan cannot be counterbalanced by greater evaporative heat loss will fans accelerate body heating. Within this context, there are physiological limitations to sweating which will impact the efficacy of fan use. Age-related decrements in sweat output are progressively observed as early as 40 years of age [27], and while very little is known about sweating responses in individuals with coronary artery disease, the decreased ability to increase cardiac output sufficiently to maintain adequate levels of skin blood flow in CAD patients, particularly those prescribed cardio-selective beta-blockers, could further compromise heat dissipation.

An initial study in young healthy adults (20-30 years of age) was conducted to determine if fan use modifies the highest humidity that can be tolerated before increases in cardiovascular (i.e., heart rate) and thermal (i.e. core temperature) strain are observed. Compared to no fan use, the relative humidity (RH) at which cardiovascular strain occurred was ~20% RH higher with a fan at 36°C, and ~10% RH higher with a fan at 42°C (Fig. 1). Moreover, thermal strain was only observed at 36°C without a fan, and at a ~10% higher RH at 42°C with a fan (Fig. 1; right). These results demonstrate that fan use might be beneficial during heatwaves, even at high ambient temperatures of 42°C, since it delayed the point at which cardiovascular and thermal strain occurred. Since younger adults are generally not considered at risk during heat waves, we performed a follow-up study that assessed the efficacy of fan use for cooling healthy older (60-80 years) adults during a simulated heatwave at 42°C[2]. In contrast to the findings in younger adults, we found that fan use resulted in a higher heart rate and core temperature in older individuals (Fig. 2). In this case, fan use was detrimental, an observation that is likely explained by age-related reductions in sweating capacity[2,16,17].

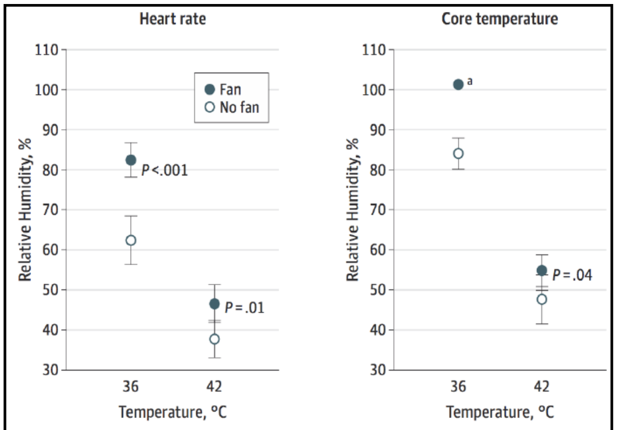

Fig 1. Relative humidity at which cardiovascular (heart rate, left) and thermal (core temperature, right) strain occur without (white circles) and with (filled circles) fan use in young adults, at 36°C and 42°C. P-values given for fan/no fan comparisons [3].

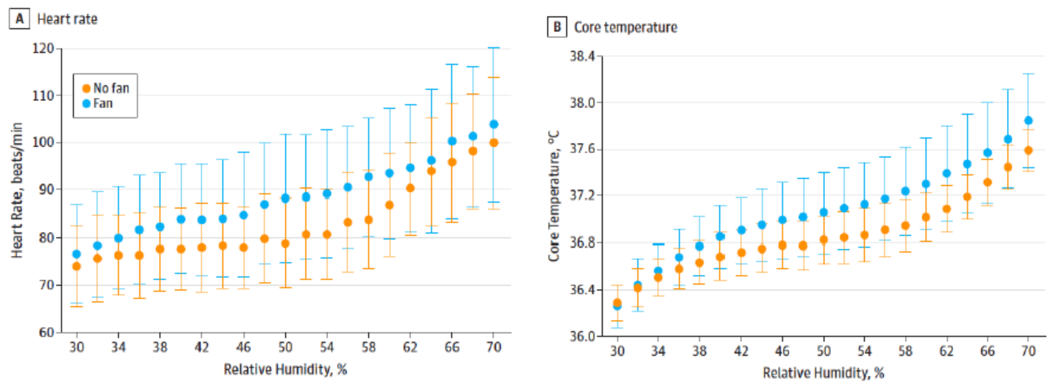

Fig 2. Heart rate (A) and core temperature (B) of older (60-80 y) adults exposed to an air temperature of 42°C with increasing relative humidity, with (blue) and without (orange) fan use [2].

Two key findings emerge from these studies; a) fan use has clear protective potential in young healthy people even at temperatures that far exceed the current public health guidelines (e.g. WHO); and b) the efficacy of fan use for cooling is dependent on the ability to physiologically wet the skin and as such fans become detrimental when used alone in older individuals because of impairments in sweating that occur with ageing[28,29]. It stands to reason, however, that age-related reductions in sweating can be compensated in older individuals by simply applying water to the skin surface. This would in turn greatly increase the cooling effect of fan use. Since heat loss capacity will likely be most limited in CAD patients prescribed beta-blockers secondary to a blunted skin blood flow, the benefits of skin wetting with fan use may be most pronounced in this group during heatwave conditions. However, the efficacy of skin wetting with or without fan use

to alleviate thermal and/or cardiovascular strain of CAD patients during heat waves has not been evaluated.

3- Objectives and evaluation criteria

3.1. Primary objective

The primary objective of this study is to identify the optimal cooling strategy to alleviate cardiovascular strain of CAD patients exposed to typical North American heatwave conditions (38°C with 60% RH). Cardiovascular strain will be quantified using rate pressure product, calculated as heart rate × systolic blood pressure. To address this objective, we will test the following hypothesis: During simulated heatwave conditions of 38°C and 60% RH, a control (no intervention) condition will result in a greater increase in rate pressure product compared to conditions of: fan use alone; skin wetting alone; fan use + skin wetting.

3.2. Secondary objective

A secondary objective is to identify the optimal cooling strategy to alleviate cardiovascular strain of CAD patients exposed to typical Australian heatwave conditions (46°C with 10% RH). Cardiovascular strain will again be quantified using rate pressure product. To address this objective, we will test the following hypothesis: During simulated heatwave conditions of 46°C and 10% RH, a control (no intervention) condition will result in a greater change in rate pressure product relative to a condition of skin wetting.

The effect of fan use (with or without skin wetting) will not be evaluated under Australian heatwave conditions. Preliminary data collected in young adults and provided by collaborator Prof. Ollie Jay (University of Sydney) demonstrate that fan use during these conditions results in a greater increase in rate pressure product compared to no fan use (Fig. 3).

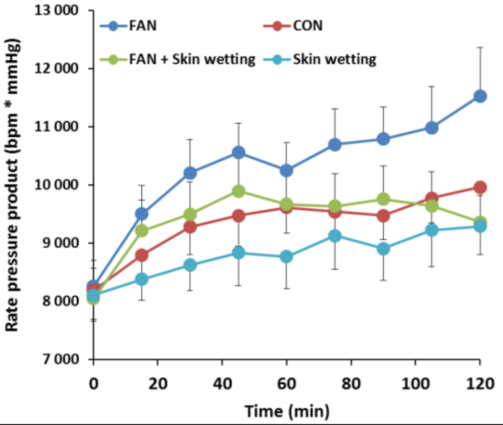

Fig 3. Rate pressure product (heart rate × systolic blood pressure) of young healthy adults (18-30 years) during a 2 hour exposure at 46°C and 10% RH. The data demonstrate that, under these conditions, fan use (FAN, dark blue) results in greater rate pressure product compared to: no intervention (CON, red); fan with additional skin wetting (FAN + skin wetting, green) and; skin wetting alone (light blue). Values are mean ± SE (n=12).

These observations can likely be explained by the fact that sweat evaporation is already maximal at such high heat and dry humidity conditions. As such, increased air velocity with fan use under these conditions greatly increases convective heat gain without resulting in greater sweat evaporation. Considering that older adults with CAD are likely to have a compromised sweating ability, we would not expect any benefit of fan use in this population during simulated heatwave conditions of 46°C and 10% RH. In fact, we would expect fan use to result in greater cardiovascular strain. For this reason, we will not evaluate the effect of fan use under these conditions.

3.3. Exploratory objectives

A tertiary objective is to examine if beta-blockers and alter the effectiveness of cooling interventions during each simulated heat wave conditions. To address this objective, we will compare a group of CAD patients prescribed beta-blockers to a group of CAD patients not taking beta-blockers. To account for the potential confounding influence of calcium channel blockers, patients prescribed calcium channel blockers will be asked to withhold this medication for 48 hours prior to each study visit.

Another tertiary objective is to evaluate how the interventions affect thermal and cardiovascular responses during each simulated heatwave condition.

3.4. Primary outcome

The primary endpoint will be the change in rate pressure product (heart rate x systolic blood pressure) from baseline (pre-exposure) to the end of a 3 hour simulated North American heatwave exposure (38°C and 60% RH).

3.5. Secondary outcome

The secondary endpoint will be the change in rate pressure product (heart rate x systolic blood pressure) from baseline (pre-exposure) to the end of a 3 hour simulated Australian heatwave exposure (46°C and 10% RH).

3.6. Exploratory outcomes

Tertiary endpoints will include, for each simulated heatwave condition, the change in rate pressure product from baseline (pre-exposure) to the end of the 3 hour simulated heatwave exposures between the group of CAD patients prescribed beta-blockers and the group of CAD patients not taking beta-blockers. Tertiary endpoints will also include: core and skin temperatures, heart rate, blood pressure, cardiac output, skin blood flow, local sweat rate, whole-body sweat rate, thermal comfort and thermal sensation.

**OBJECTIVES**	**EVALUATION CRITERIA**
Primary	
What is the optimal intervention to alleviate cardiovascular strain of CAD patients during North American heat wave conditions?	*Change in rate pressure product from baseline to the end of a 3 hour exposure at 38°C and 60% RH.*
Secondary	
What is the optimal intervention to alleviate cardiovascular strain of CAD patients during Australian heat wave conditions?	*Change in rate pressure product from baseline to the end of a 3 hour exposure at 46°C and 10% RH.*
Exploratory	
Do beta-blockers alter the effectiveness of cooling interventions during North American heat wave conditions in patients with CAD?	*Change in rate pressure product from baseline to the end of a 3 hour exposure at 38°C and 60% RH between a group of CAD patients prescribed beta-blockers vs. a group of CAD patients not taking beta-blockers.*

OBJECTIVES	EVALUATION CRITERIA
Do beta-blockers alter the effectiveness of cooling interventions during Australian heat wave conditions in patients with CAD?	*Change in rate pressure product from baseline to the end of a 3 hour exposure at 46°C and 10% RH between a group of CAD patients prescribed beta-blockers vs. a group of CAD patients not taking beta-blockers.*
How do the cooling interventions affect thermal and cardiovascular responses during simulated heatwave conditions?	*Change in the following variables during each of the heatwave exposures: core and skin temperatures, heart rate, blood pressure, cardiac output, skin blood flow, local sweat rate, whole-body sweat rate, thermal comfort and thermal sensation.*

5- Methods

5.1 Study design

Participants will be asked to volunteer for 1 preliminary visit, 1 fasting blood sample visit and 6 study visits. Participants will first undergo the 4 study visits for the simulated North American heatwave condition to minimize attrition for the primary objective. Participants will subsequently undergo the 2 study visits for the simulated Australian heatwave condition. Within each heatwave condition (North American, Australian) the sequence under which participants will undergo the study visits will be randomized. Prior to each visit, participants will be asked to refrain from strenuous physical activity, alcohol, and caffeine for 12 h. To ensure there is no carry-over effect, study visits will be performed on separate days, with at least 72 h between visits. Participants prescribed calcium channel blockers will be asked to withhold this medication for 48 hours prior to each study visit.

During the preliminary visit, participants will be invited to the laboratory and will be given the chance to familiarize themselves with all of the procedures and equipment involved in the study. They will also be given time to read the consent form and ask any questions they may have regarding their participation in the study. If they agree to participate, they will be asked to sign the consent form. Participants will then be asked to fill out a medical history questionnaire, following which we will measure their body weight and height, their resting heart rate and rhythm, and their resting blood pressure. A fasting blood sample will also be drawn.

During the study visits, participants will enter an environmental chamber maintained at either 38°C and 60% RH or 46°C and 10% RH. For each visit, the participant will remain within the environmental chamber and will rest in a seated position for 3 hours. The 6 study visits will consist of the following:

1) 38°C and 60% RH, no intervention (control)
2) 38°C and 60% RH, with fan use
3) 38°C and 60% RH, with skin wetting
4) 38°C and 60% RH, with fan use and skin wetting
5) 46°C and 10% RH, no intervention (control)
6) 46°C and 10% RH, with skin wetting

5.2 Description of the population

Participants will be male or female, aged between 50 and 80 years of age, with stable coronary artery disease.

5.2.1 Inclusion criteria

- History of angiographic coronary disease (≥70% arterial diameter narrowing of at least one major epicardial coronary artery) and/or prior coronary revascularization and/or documented prior acute coronary syndrome and/or stable angina and/or perfusion defect during exercise testing.
- No CAD-related hospitalisations or changes in cardiac medications or change in pattern of angina for at least 3 months prior to enrolment.

5.2.2 Exclusion criteria

- Body mass index ≥35 kg/m^2.
- Currently undertaking estrogen therapy.
- Evidence of current fluid and electrolyte disorders, anemia, abnormal thyroid function, arrhythmias, diabetes, renal disease, liver disease, cerebrovascular disease, significant pulmonary disease, endocrine abnormalities, significant cognitive impairment, psychiatric disorder, substance abuse, degenerative neurological condition or any other medical condition deemed to pose risk during the proposed experiments.
- Uncontrolled hypertension (>180/110 mmHg).
- Recent (<3 months) coronary bypass surgery.
- Ejection fraction <40% and/or clinical evidence/history of heart failure.
- Significant valvular heart disease
- Resting ECG abnormalities interfering with observation of ST segment changes during testing.

5.2.3 Strategy for recruiting and retaining participants in the study

Potential participants will be recruited through the following strategies:

1) Approved advertisements. The advertisements will instruct interested individuals to contact, by phone or email, a member of the research team.
2) Phone calls to previous participants which gave their consent to be contacted for new research studies.
3) Phone calls to potential participants which gave their consent to be contacted about research studies during their annual medical visit.
4) Direct contact with patients enrolled in the ÉPIC prevention clinics. A member of the research staff will regularly attend the ÉPIC prevention clinics to provide information regarding the study. Interested participants will be given a copy of the approved advertisements and will be instructed to contact, by phone or email, a member of the research team

Once an interested individual contacts us, a member of the research staff will assess their eligibility to participate in the study by phone. If interested individuals meet the inclusion criteria, they will be invited to visit the laboratory for a preliminary visit. During this visit, a member of the research staff will familiarize interested individuals with all of the procedures and measurements of the study. Interested individuals will be given the opportunity to ask questions and they will also be given the time they need to read the consent form. If they decide to participate in the study, they will be asked to sign the consent form and fill out a medical history. Final confirmation of their eligibility to participate in the study will be determined by measuring their resting heart rate/rhythm and blood pressure and obtaining the results of a fasting blood sample.

5.3 Research protocol

5.3.1 Description of the visits

Upon awakening the day of a study visit, participants will be asked to swallow an ingestible telemetric pill which will have been provided to them beforehand. Upon arrival to the laboratory, participants will be asked to provide a urine sample and weigh themselves nude (in private). Participants will then be instrumented following which they will rest in the seated position for a 10 min baseline period at a room temperature of 24°C. Following this baseline period, they will enter an environmental chamber maintained at either 38°C and 60% RH or 46°C and 10% RH and will be seated within a semi-recumbent mesh chair that will allow for convective airflow. Participants will remain seated for 3 hours within the environmental chamber while the relevant cooling intervention is administered. During the exposure, heart rate will be measured continuously from an ECG signal and blood pressure measurements will be taken every 20 minutes by automated auscultation of the brachial artery. Rate pressure product (**RPP**) will subsequently be calculated at baseline and every 20 minutes during the 3 hour exposure (see arrows in timeline below). Participants will be provided with 200 ml of water every 20 minutes. At the end of the 3 hour period, participants will exit the environmental chamber to be de-instrumented and will be asked to weigh themselves nude in private to calculate whole-body sweat rate. During the study visits with fan use, an 18" diameter fan will face the participant from a distance of 1.0 m to provide an airflow of 4.0 m/s. During the study visits with skin wetting, tap water (~18°C) will be applied every 10 minutes to the forehead, face, arms and neck using a cloth.

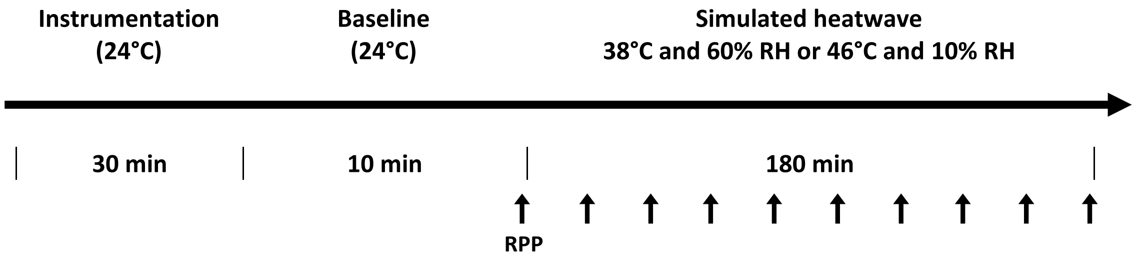

5.4 Measurements

1) Body weight will be measured during the preliminary visits, as well as before and at the end of each study visit with a scale.

2) Body height will be measured during the preliminary visit with a stadiometer.

3) Blood samples will be drawn from a sterile catheter inserted into a peripheral vein.

4) Heart rhythm will be acquired during the preliminary visits, as well as continuously during the study visits from an ECG signal. Heart rate will also be measured continuously during the study visits with a monitor placed around the chest.

5) Blood pressure will be measured during the preliminary visits, as well as every 20 minutes during the study visits by automated auscultation of the brachial artery.

6) Internal body temperature will be measured continuously during the study visits with an ingestible telemetric pill.

7) Oral temperature will be measured by placing an oral thermometer under the tongue.

8) Skin temperatures will be measured continuously during the study visits by taping sensors to the skin surface.

9) Local sweat rate will be measured continuously during the study visits using plastic capsules attached to the skin surface and ventilated with dry air.

10) Skin blood flow will be measured continuously during the study visits on the forearm by laser-Doppler flowmetry.

11) Cardiac output will be measured every 20 minutes during the study visits with an inert gas rebreathing technique.

12) Urine specific gravity (as a measure of hydration status) will be determined from a urine sample collected at the beginning of each study visit.

13) Thermal comfort and sensation will be measured every 20 minutes during the study visits using visual scales.

Measurements	Preliminary visit	Laboratory visits
Information and consent	X	
Familiarization	X	
Medical history	X	
Body height	X	
Blood sample	X	X
Body weight	X	X
Heart rate and rhythm	X	X
Blood pressure	X	X
Internal body temperature		X
Oral temperature		X
Skin temperatures		X
Local sweat rate		X
Skin blood flow		X
Cardiac output		X
Urine specific gravity		X
Thermal comfort and sensation		X

6- Statistics

6.1 Sample size

The sample size computation is based on comparison of the primary endpoint (change in rate pressure product from baseline to the end of a 3 hour simulated heatwave exposure to 38°C and

60% RH) between the 4 conditions of control, fan use alone, skin wetting alone, fan use + skin wetting.

The mean and standard deviation of the change in rate pressure product were taken from the preliminary data presented in Figure 3 and are expected to range between 1000 bpm x mmHg and 1700 bpm x mmHg for the mean and between 1700 bpm x mmHg and 2000 bpm x mmHg for the standard deviation. The standard deviation of the difference between conditions is expected to be similar.

The main interest is to compare the control condition with the 3 other conditions taken individually, which implies a significance level of 0.0167 (0.05 / 3). A difference of 1250 bpm x mmHg in change in rate pressure product between the control condition and any other condition is judged to be clinically relevant.

In this context a sample size of 30 participants will give 80% power to detect a difference of 1250 bpm x mmHg in change between the control condition and any other condition, assuming that the standard deviation of the difference between conditions is 2000 bpm x mmHg and using a paired t-test with a 0.0167 two-sided significant level. To account for an approximately 10% loss to follow-up rate, 34 participants will be included in the study. Of the 34 participants, 17 will be taking beta-blockers whereas the other 17 will not be taking beta-blockers.

6.2 Statistical analysis

Study variables will be presented using descriptive statistics. Mean, standard deviation, median, minimum and maximum will be presented for continuous variables. Number and percentage will be presented for categorical variables. Statistical assumptions underlying planned statistical tests will be examined and data transformation or non-parametric analysis might be used if appropriate. All statistical tests will be conducted at the 0.05 significance level unless specified otherwise.

<u>Primary analysis:</u> A one-way repeated analysis of variance (ANOVA) model will be used to compare the primary endpoint (change in rate pressure product under the 38°C and 60% RH heatwave exposure) between the conditions of: control, fan alone, skin wetting alone and fan + skin wetting. Contrasts under this model will allow for the three main comparisons:

- Control vs. fan alone
- Control vs. skin wetting alone
- Control vs. fan + skin wetting

These three comparisons will be tested at the 0.0167 significance level to control for the multiplicity of tests. This will be considered as the primary analysis of the study.

Since rate pressure product will be measured several times during the 3 hour simulated heatwave exposure, a two-way repeated measures ANOVA model on rate pressure product, having a term for condition, a term for time and a term for the condition x time interaction, might also be used to more precisely describe the pattern of change over the 3 hours of heat exposure across the conditions.

Secondary analysis: The change in rate pressure product from start to end of a 3 hour simulated heatwave exposure at 46°C and 10% RH will be compared between the control and skin wetting conditions using a paired t-test.

Exploratory analyses: To study the impact of beta-blockers, repeated measures ANOVA models on change in rate pressure product with a term for condition, a term for beta-blockers (yes/no) and a term for the interaction between condition and beta-blockers will be used for each heatwave condition. Other tertiary endpoints measured throughout the 3 hour exposures will be analyzed for each heatwave condition with a two-way repeated measures ANOVA model, having a term for condition, a term for time and a term for the condition x time interaction.

7- References

1. Meehl GA, Tebaldi C. More intense, more frequent, and longer lasting heat waves in the 21st century. *Science (New York, NY)* 2004;305:994-7.

2. Gagnon D, Romero SA, Cramer MN, Jay O, Crandall CG. Cardiac and Thermal Strain of Elderly Adults Exposed to Extreme Heat and Humidity With and Without Electric Fan Use. *JAMA* 2016;316:989-91.

3. Ravanelli NM, Hodder SG, Havenith G, Jay O. Heart rate and body temperature responses to extreme heat and humidity with and without electric fans. *JAMA* 2015;313:724-5.

4. Robine JM, Cheung SL, Le Roy S, Van Oyen H, Griffiths C, Michel JP, Herrmann FR. Death toll exceeded 70,000 in Europe during the summer of 2003. *C R Biol* 2008;331:171-8.

5. World Health Organization. Heat-Health Action Plans2008.

6. World Health Organization. Public health advice on preventing health effects of heat2011.

7. World Meteorological Organization, World Health Organization. Heatwaves and Health: Guidance on Warning-System Development2015.

8. United States Environmental Protection Agency. Excessive heat events guidebook2006.

9. Centers for Disease Control and Prevention. Extreme Heat Prevention Guide. 2006. (Accessed November 24, 2016, at https://www.cdc.gov/disasters/extremeheat/heat_guide.html, accessed November 24, 2016.)

10. Health Canada. Extreme heat events guidelines: Technical guide for health care workers. Ottawa, Ontario: Water Air and Climate Change Bureau, Healthy Environments and Consumer Safety Branch; 2011.

11. Institut national de prevention et d'éducation pour la santé (France). Canicule et fortes chaleurs : agir pour prévenir les risques. 2015. at http://inpes.santepubliquefrance.fr/10000/themes/evenement_climatique/canicule/canicule-agir.asp, accessed November 24, 2016.)

12. Centers for Disease Control and Prevention. Heat-Related Deaths - United States, 1999-2003. *Morbidity and Mortality Weekly Report (MMWR)* 2006;55:796-8.

13. Semenza JC, McCullough JE, Flanders WD, McGeehin MA, Lumpkin JR. Excess hospital admissions during the July 1995 heat wave in Chicago. *Am J Prev Med* 1999;16:269-77.

14. Semenza JC, Rubin CH, Falter KH, Selanikio JD, Flanders WD, Howe HL, Wilhelm JL. Heat-related deaths during the July 1995 heat wave in Chicago. *The New England journal of medicine* 1996;335:84-90.

15. Kenney WL, Craighead DH, Alexander LM. Heat waves, aging, and human cardiovascular health. *Med Sci Sports Exerc* 2014;46:1891-9.

16. Gagnon D, Crandall CG. Electric fan use during heat waves: Turn off for the elderly? *Temperature (Austin)* 2017;4:1-3.

17. Gagnon D, Romero SA, Cramer MN, Kouda K, Poh PY, Ngo H, Jay O, Crandall CG. Age Modulates Physiological Responses during Fan Use under Extreme Heat and Humidity. *Med Sci Sports Exerc* 2017;49:2333-42.

18. Hajat S, O'Connor M, Kosatsky T. Health effects of hot weather: from awareness of risk factors to effective health protection. *Lancet* 2010;375:856-63.

19. Kenny GP, Yardley J, Brown C, Sigal RJ, Jay O. Heat stress in older individuals and patients with common chronic diseases. *CMAJ* 2010;182:1053-60.

20. Gordon NF, van Rensburg JP, Russell HM, Kawalsky DL, Celliers CP, Cilliers JF, Myburgh DP. Effect of beta1 selective adrenoceptor blockade on physiological response to exercise. *Br Heart J* 1985;54:96-9.

21. Gordon NF, Myburgh DP, Schwellnus MP, van Rensburg JP. Effect of beta-blockade on exercise core temperature in coronary artery disease patients. *Med Sci Sports Exerc* 1987;19:591-6.

22. Sheridan SC. A survey of public perception and response to heat warnings across four North American cities: an evaluation of municipal effectiveness. *Int J Biometeorol* 2007;52:3-15.

23. Salamanca F, Georgescu M, Mahalov A, Moustaoui M, Wang M, Svoma BM. Assessing summertime urban air conditioning consumption in a semiarid environment. *Environ Res Lett* 2013;8:034022.

24. Salamanca F, Georgescu M, Mahalov A, Moustaoui M, Wang M. Anthropogenic heating of the urban environment due to air conditioning. *J Geophys Res Atmos* 2014;119:5949-65.

25. Bernard SM, McGeehin MA. Municipal heat wave response plans. *Am J Public Health* 2004;94:1520-2.

26. Gupta S, Carmichael C, Simpson C, Clarke MJ, Allen C, Gao Y, Chan EY, Murray V. Electric fans for reducing adverse health impacts in heatwaves. *Cochrane Database Syst Rev* 2012;7:CD009888.

27. Larose J, Boulay P, Sigal RJ, Wright HE, Kenny GP. Age-related decrements in heat dissipation during physical activity occur as early as the age of 40. *PLoS One* 2013;8:e83148.

28. Inoue Y, Nakao M, Araki T, Murakami H. Regional differences in the sweating responses of older and younger men. *J Appl Physiol (1985)* 1991;71:2453-9.

29. Inoue Y, Shibasaki M. Regional differences in age-related decrements of the cutaneous vascular and sweating responses to passive heating. *Eur J Appl Physiol Occup Physiol* 1996;74:78-84.

Final protocol – USyd 2018-496: Use of fans and/or skin-wetting as a low cost cooling strategy for older adults during heatwaves

Funding organizations
National Health and Medical Research Council

Principal investigator
Ollie Jay (PhD)

Collaborators
Yorgi Mavros, PhD
Maria Fiatarone Singh, PhD
Lily Hospers
Amy Harwood, PhD
Nicole Vargas, PhD

Date of initial protocol approval (Version 01)	29-08-2018
Modification to existing approved application (Version 02)	11-09-2018
Modification to existing approved application (Version 03)	15-10-2018
Modification to existing approved application (Version 04)	19-11-2018
Change in personnel only	22-08-2019
Modification to existing approved application (Version 05)	01-11-2019
Change in personnel only	14-09-2020
Modification to existing approved application (Version 06)	16-10-2020
Modification to existing approved application (Version 07)	03-03-2021
Modification to existing approved application (Version 08)	19-05-2021
Change in personnel only	08-06-2021
Modification to existing approved application (Version 09)	16-06-2021
Modification to existing approved application (Version 10)	01/12/2021
Change in personnel only	20-07-2023

1- Outline the theoretical, empirical and/or conceptual basis, background evidence for the research proposal with relevant literature.

The frequency, intensity and duration of heat waves are increasing globally (Bi et al. 2011), and with these increases we can expect with a high level of certainty a parallel rise in heat-related morbidity and mortality (Hajat, O'Connor & Kosatsky, 2010). In an attempt to combat this, there is a need for evidence-based guidelines to be made available that educate people on the steps they can take to protect themselves during extreme heat events. One particular population group at a disproportionately higher risk of morbidity and mortality during these extreme weather events is the elderly (Hajat, O'Connor & Kosatsky, 2010). Therefore, it is of great importance to make targeted recommendations for this population.Air-conditioning is the best protection during extreme heat events, but it is not accessible to many of the most vulnerable populations and widespread use and reliance on it during extreme heat events can cause blackouts (Kaiser et al., 2001; Semenza et al., 1996). Researchers began examining the effectiveness of using a fan as a cooling intervention and found that public health recommendations regarding fan-use were not uniform between agencies and often not evidence-based (Jay et al., 2015). If fan-use were to be found effective, it would provide a low-cost solution with a low-energy requirement. Research to date suggests that the benefits of fans have been greatly underestimated by public health agencies (Jay et al., 2015). Despite organizations such as the WHO, EPA and the Government of Victoria advising against using fans in hot conditions, a study published in the Journal of the American Medical Association (JAMA) demonstrated that fan use reduced physiological strain in young healthy men in simulated hot/humid heat wave conditions (Ravanelli et al., 2015). The use of a fan increased airflow which improved sweating efficiency and led to greater evaporative heat loss. The effectiveness of fan-use does depend on both the unique environmental conditions and the individual; it has been suggested that in certain conditions fans may be less effective and even harmful to older adults due to age-related decrements in sweating (Gagnon et al., 2016). Due to the findings to date it is clear that there is a need for evidence based recommendations specific to both the environmental conditions and population-specific, particularly those populations who have compromised thermoregulatory ability, such as the elderly. In a recent article by Jay and Capon (2017) it was proposed that external skin wetting could be used by the elderly to compensate for their decreased sweating ability, while still gaining the benefits of increased latent heat loss as a result of improved evaporative efficiency with fan-use. This combination has the potential to be a simple, cost-effective cooling intervention that has 50-times lower cost and electricity requirement than air conditioning and so therefore can be utilized by vulnerable populations in a low-resource environment. We propose the below study to examine if fan-use, in combination with skin-wetting can be an effective cooling strategy that is able to decrease the physiological strain experienced by elderly during heat waves, and as a result decrease their chance of experiencing an adverse cardiovascular event. During the 2003 heat wave in Lyon, France, antihypertensive use was associated with a 177% increased risk of mortality in adults presenting to the emergency department with heat stroke (Argaud et al., 2007). Due to this historical evidence and the prevalence of hypertension in older adults, it is important to include older adults on antihypertensives in our study to adequately represent the older adult population, while also investigating the impact of antihypertensive use on individuals' responses to simulated heat wave exposure and the proposed interventions. We believe the proposed project will provide evidence

that will help public health and government organizations make evidence-based environment and population specific recommendations for older adults during heatwaves.

2- Outline the methodology for the research proposal including:

2.1. Research Questions:

The primary research question guiding this project is: Are fans and/or skin wetting effective in decreasing the physiological strain experienced by elderly during a simulated hot humid heatwave (38°C with 60% relative humidity).

The secondary research question guiding this project is: are fans and/or skin-wetting effective in decreasing the physiological strain experienced by elderly during a simulated hot, dry heatwave (45°C with 15% relative humidity).

2.2 Research hypotheses:
The research hypotheses for this study are:

3. In the hot and humid condition, all interventions will result in improved cooling compared to the control (no-fan) trail. With fan and skin-wetting (FSW) being the most effective, then no fan and skin-wetting (NFSW), then fan (F), and finally, the control, no-fan (NF).
4. In the very hot and dry condition, skin wetting with no fan (SWNF) will be the most effective cooling intervention followed by fan and skin-wetting (FSW). We expect the fan (F) intervention to be less effective than the control, no-fan (NF).

4.3 *Participant characteristics:* To complete this study, 56 (greater than or equal to 60 years) adults will be recruited from previous participants who have consented to being contacted for future studies and through the distribution of recruitment flyers at both the Cumberland and Camperdown campuses at The University of Sydney.

4.4 *Sample size:* The sample size computation is based on comparison of the primary endpoint (change in rate pressure product from baseline to the end of a 2 h simulated heatwave exposure to 38ºC and 60% relative humidity) between the 4 conditions of control, fan use alone, skin wetting alone, fan use + skin wetting. This preliminary data from young healthy participants was provided by Nate Morris, a former PhD student from within our laboratory. A non-repeated measures ANOVA on this data demonstrates 20% (eta squared of 0.2) of the variability was due to the intervention. Using GPower 3.1.9.2 software, an ANCOVA: Fixed effects, main effects and interactions f-test was performed using an alpha of 0.05, beta of 0.1 and an effect size of 0.5 to determine a total sample size of 45 is needed to obtain sufficient statistical power. To account for approximately 20% loss to follow up, 56 participants will be included in the study. Of the 56 participants, 14 will be healthy older females, 14 will be healthy older males, 14 will be healthy females taking antihypertensive medication, and 14 will be older males taking antihypertensive medication.

4.5 *Inclusion/Exclusion Criteria:*
Inclusion Criteria: Non-smokers or ex-smokers (greater that or equal to 1y since quitting), fluent/have no difficulty understanding and speaking English, and age of greater than or equal

to 60 y. Age will be matched across all groups. Participants with hypertension must have been diagnosed as hypertensive and taking antihypertensive medications.

Exclusion criteria: body mass index greater than or equal to 35. No history/signs/symptoms of coronary artery disease or heart failure. Currently undertaking hormone replacement therapy. Currently taking medications known to cause hyper- or hypo-hidrosis (except those taken for hypertension). Currently taking a beta-blocker. Evidene of current fluid and electrolyte disorders, anemia, abnormal thyroid function, arrhythmias, diabestes, renal disease, liver disease, cerebrovascular disease, significant pulmonary disease, endocrine abnormalities, uncontrolled hypertension (180/110mmHg), significant cognitive impairment, psychiatric disorder, substance abuse, degenerative neurological condition or any other medical condition deemed to pose risk during the proposed testing or experiments, or preclude them from completing the screening stress test.

4.6 *Data collection protocol:*

The preliminary sessions and the 4 experimental sessions (with the option of doing an additional 4 experiemental trials) will take place at the University of Sydney, Susan Wakil Health Building, Camperdown Campus. The time involvement will be approximately 1 hour for the first preliminary session, and 4 hours for each of the experimental sessions. This involves a total time commitment of approximately 16 hours (or approximately 33 hours if the participant chooses to do an additional four trials).We are recruiting participants for at least four experimental sessions that will take place in the hot, humid condition and providing them with the option of doing an additional four experimental sessions that will be carried out under a different simulated heatwave condition (very hot, dry). These two simulated heatwaves provide very different interactions between the individual, the environment and the proposed interventions and so it is of great importance to study both types. For example, in a previous study carried out in our lab a fan was beneficial in hot-humid conditions due to the increased airflow increasing sweating efficiency and therefore evaporative heat loss, whereas in the hot-dry condition the increased airflow accelerated dry heat gain and so fan-use was actually unfavourable. We understand that the one preliminary sessions and 8 experimental sessions are a big time commitment and so we have chosen to take the above approach to minimize attrition for the primary objective. Investigating the low cost at-home cooling interventions in the hot, humid condition is our primary objective as these are the types of heatwaves most common worldwide. Investigating the same interventions in the very hot, dry condition allows us to investigate their impact in the type of heatwave most prevalent in Australia.

Pre-screening and preliminary sessions: Participants will be initially screened over the phone using a standardized phone screen based on the inclusion and exclusion criteria of our study. If the participants pass the phone screen they will be invited in for a preliminary session. The first preliminary session involves a complete medical history, physical examination and cardiac stress test. This screening will be carried out by geriatrician Maria Fiatarone Singh, MD. The following equipment and techniques will be used:

•Cardiac Stress Test: The participant will undergo a physician-supervised graded exercise test on a treadmill while undergoing ECG and blood pressure monitoring. Both are described below.

•Venepuncture: If determined necesary by the study physician, a blood sample will obtained by peripheral puncture of the forearm vein during their second preliminary session. Venepuncture will be performed by personnel with phlebotomy training.

If the participant agrees to participate in this study, they will undergo testing at the Camperdown Campus of the University of Sydney for approximately 4 hours on 4 separate occasions. (There is an option to participate in an additional four experimental sessions.) Each experimental session will be separated by at least 72 hours. Participants will be asked to abstain from alcohol and caffeine, avoid strenuous exercise in the 12 h prior to each experimental session, and will be instructed to consume a light meal and 0.5L of water ~2 h before arriving at the lab. They will be provided with a standardized singlet and shorts to wear and will undergo 30-min of baseline rest (seated) in a thermoneutral room (~24°C), where they will be instrumented using the equipment described below. After 30-min, they will then enter a climate-controlled chamber, where they will be seated unrestrained in a standard armchair for the remainder of the 3-hour experimental protocol. This protocol will consist of the participant sitting in a 38ºC and 60% relative humidity room. (If they choose to participate in the additional four trials the conditions will be 45ºC and 15% relative humidity). During each session the participant will undergo one of the following four conditions: no cooling intervention, use of an electric fan, self-wetting the skin with a sponge, or use of an electric fan while self-wetting the skin with a sponge. Additionally, in all trials they will be provided with 3ml per kg of cold (~18ºC) water every hour. In all fan trials, they will be seated 1.0 m from an 18” diameter fan set to the highest setting. The following equipment and techniques will be used:

•Rectal temperature sensor: The participant will be asked to insert a flexible sensor 10-12cm into their rectum. A marker is placed on the sensor using sterile surgical tape. The participant will insert the sensor until the tape reaches their anal surface. The insertion of the sensor may cause some mild discomfort and minor irritation; however, this sensation soon passes. The participant will receive proper instruction regarding the placement of the sensor to ensure their safety and comfort. The participant will be responsible for the insertion of this sensor. It will provide the researcher with an indication of the amount of heat stored in their body and will be tracked throughout the entirety of each experimental session

•Skin temperature sensors: Eight skin sensors will be taped to the participants skin surface with hypoallergenic tape. Some hair may need to be shaved (by the use of disposable razors) in order to secure the probes adequately. These probes give an indication of skin temperature and heat loss from the skin and will be recorded throughout the entirety of each experimental session.

•Blood pressure: An automated blood pressure monitor will be strapped to the participants arm and blood pressure will be taken every fifteen minutes during the experimental protocol and will be used by researchers to better characterize skin and limb blood flow and calculate RPP (heart rate x systolic blood pressure). Blood pressure will also be taken three times during the orthostatic intolerance test described below.

•ECG monitoring: 12 soft electrodes will be stuck to the participants torso and will measure the electrical signals of their heart. This will produce signals on a display that will be continuously monitored to ensure the participants safety throughout the cardiac stress test during the first preliminary session and each experimental session.

•Heart rate: Heart rate will be measured using the ECG monitoring.

•Skin blood flow: A flexible laser probe will measure skin blood flow non-invasively at the upper back during the entirety of each experimental session. This measurement device does not result in any discomfort or residual medical effects.

•Limb blood flow: Limb blood flow will be determined using venous occlusion plethysmography. This technique involves a rapidly inflating cuff around the participants arm to a pressure of ~60 mmHg (compared to ~180 mmHg during a blood pressure reading). A strain gauge that resembles an elastic band will be placed on their forearm and this will record minor changes in limb volume. This measurement will be taken every thirty minutes during each experimental session.

•Ventilated sweat capsules: A small plastic capsule connected to plastic tubing will be placed on the participants upper back and forearm. Dry air is passed through this capsule and a humidity sensor will pick up humidity from the skin and provides a measurement of local sweat rate throughout each experimental session.

•Cognitive test: The participant will be asked to complete a short-form of the Stroop Colour and Word Test (SCWT) immediately before and at the completion of the heatwave exposure. This test involves them looking at various words and identifying which color the words are shown in.

•Whole-body sweat loss: The participant will be weighed on a platform scale immediately before and at the completion of the heatwave exposure in order to compare whole-body sweat losses.

•Rating of thermal comfort and sensation: The participant will be asked to rate how warm they feel and how uncomfortable the heat makes them on two separate visual analogue scales every fifteen minutes during each experimental session.

•Nausea and lightheadedness scale: The participant will be asked to rate how much (if any) nausea or lightheadedness they are experiencing and tick if they are experiencing any other symptoms (including paleness, muscle cramps, tiredness, weakness and headache) every fifteen minutes during each experimental session.

•Orthostatic hypotension: At the completion of the heatwave exposure the participants blood pressure will be taken. They will then be asked to stand up and their blood pressure will be taken after 1 minute of standing and then again after 3 minutes of standing. This test is carried out to see if there is evidence of postural hypotension (a drop in blood pressure within 3 minutes of standing from a seated position).

All equipment and procedures described above are commonly used procedures and have been used multiple times by the investigators conducting the study (1-4, 9, 10) as well as research groups (5-8) in the field of thermoregulation across the world and these methods are all in accordance with the Declaration of Helsinki.

4.7 Statistical Analysis:

The primary research outcomes for this study are rectal temperature (Tre), rate pressure product (RPP), heart rate (HR), blood pressure (BP), thermal sensation (TS), thermal comfort (TC) and pre to post trial whole-body sweat losses (WBSL).Secondary outcomes to be compared are skin temperature (TSk), limb blood flow (LBF), skin blood flow (SkBF), local sweat rate (LSR), cognitive performance, rating of nausea and light headedness (NL), and evidence of orthostatic hypotension (OH).All data will be compared between all four interventions and both conditions (very hot and dry, and hot and humid).To assess the primary and secondary outcome variables, pre to post trial changes as well as the means of the dependent variables will be analyzed using one-way repeated measures ANOVAs with the repeated factor of cooling intervention (four levels: no fan (NF), fan (F), fan and skin-wetting (FSW), and no fan and skin-wetting (NFSW)) will be employed. If significant main effects or interactions are found, independent differences will be assessed using a two-tailed paired Student's t-tests while maintaining a fixed probability (5%) of making a type I error using a Holm-Bonferroni correction. All statistical analyses will be performed with GraphPad Prism (version 6.0, GraphPad Software, La Jolla, CA).

3- References

1. Argaud, L., Ferry, T., Le, Q. H., Marfisi, A., Ciorba, D., Achache, P., ... & Robert, D. (2007). Short-and long-term outcomes of heatstroke following the 2003 heat wave in Lyon, France. Archives of internal medicine, 167(20), 2177-218

2. Bi, P., Williams, S., Loughnan, M., Lloyd, G., Hansen, A., Kjellstrom, T., ... & Saniotis, A. (2011). The effects of extreme heat on human mortality and morbidity in Australia: implications for public health. Asia Pacific Journal of Public Health, 23(2_suppl), 27S-36S.

3. Gagnon, D., Romero, S. A., Cramer, M. N., Jay, O., & Crandall, C. G. (2016). Cardiac and thermal strain of elderly adults exposed to extreme heat and humidity with and without electric fan use. Jama, 316(9), 989-991

4. Hajat, S., OConnor, M., & Kosatsky, T. (2010). Review: Health effects of hot weather: from awareness of risk factors to effective health protection. The Lancet, 375856-863. doi:10.1016/S0140-6736(09)61711-6

5. Jay, O., & Capon, A. (2018). Use of physiological evidence for heatwave public policy. The Lancet Planetary Health, 2(1), e10.

6. Jay, O., Cramer, M. N., Ravanelli, N. M., & Hodder, S. G. (2015). Should electric fans be used during a heat wave. Applied ergonomics, 46, 137-143

7. Kaiser R, Rubin CH, Henderson AK, Wolfe MI, et al. Heat-related death and mental illness7.during the 1999 Cincinnati heat wave. Am J Foren Med Path. 2001;22(3):303-7

8. Ravanelli, N. M., Gagnon, D., Hodder, S. G., Havenith, G., & Jay, O. (2017). The biophysical and physiological basis for mitigated elevations in heart rate with electric fan use in extreme heat and humidity. International journal of biometeorology, 61(2), 313-323.

9. Ravanelli, N. M., Hodder, S. G., Havenith, G., & Jay, O. (2015). Heart rate and body temperature responses to extreme heat and humidity with and without electric fans. Jama, 313(7), 724-725.

10. Semenza JC, Rubin CH, Falter KH, Selanikio JD, Flanders WD, Howe HL, et al. Heat-related11.deaths during the July 1995 heat wave in Chicago. New Engl J Med. 1996;335(2):84-90.

Final protocol – MHI 2019-2425: Identifying optimal cooling strategies for coronary artery disease patients during heatwaves

Funding organizations
National Health and Medical Research Council

Principal investigator
Daniel Gagnon (PhD)

Collaborators
Martin Juneau (MD)
Anil Nigam (MD)
Céline Ferland (Inf.)
Julie Lalongé (R.A.)
Georgia Chaseling (PhD)

Date of initial protocol approval (Version 01)	07-09-2018
Date of protocol amendment (Version 02)	01-21-2019
Date of protocol amendment (Version 03)	04-04-2019
Date of protocol amendment (Version 04)	09-04-2019

1- Introduction and definition of research problem

Periods of extreme heat have been on the increase for several years now. In 2004, it was predicted that these climatic events would occur more frequently, be more intense and last longer[1]. Periods of extreme heat can have catastrophic consequences for human health and well-being. The most striking example is the heatwave that hit Europe in 2003, causing the death of 70,000 people, including 40,000 in the space of 2 weeks[4]. Since, many public health agencies have developped action plans to deal with periods of extreme heat, in recognition of the danger posed by such events[5-11].

Several studies have assessed the risk factors leading to hospitalisation and death during periods of extreme heat. It is now well established that the elderly are most at risk of hospitalisation and death during such events[12-14]. However, it is important to consider that hospitalisations and deaths of elderly people during periods of extreme heat are not directly caused by excessive body hyperthermia (e.g., heatstroke). Rather, they are primarily driven by adverse cardiovascular events[15]. These observations can be explained by the cardiovascular demands imposed by heat exposure. During heat exposure, vasodilation of cutaneous blood vessels promotes heat exchange between the body and the environment. However, this vasodilation results in a fall of peripheral resistance, which must be compensated for by an increase in cardiac output to maintain blood pressure. The cardiovascular demands imposed by heat exposure could exceed the ischaemic threshold of people with known or silent coronary artery disease, predisposing them to a risk of adverse cardiovascular events even if body temperature does not reach a level associated with, for example, heatstroke.

Several studies have sought to better understand the effects of human ageing on heat loss responses (sweat production, cutaneous vasodilation), as well as on cardiovascular responses during heat exposure. Relatively fewer studies have attempted to identify simple and effective interventions to alleviate cardiovascular strain of older adults during extreme heat exposure. We recently published a pilot study that evaluated the impact of fan use on thermal (core temperature) and cardiovascular (heart rate) strain of healthy older adults (60-80 years of age)[2]. A previous study showed that the fan use reduced thermal and cardiovascular strain of healthy young adults (20-30 years of age) during exposure to a 36°C and 42°C environment[3]. In contrast to these observations, we found that fan use resulted in greater thermal and cardiovascular strain in healthy older adults during a exposure to a 42°C environment. We believe that this observation is explained by the fact that fan use does not result in a net improvement in heat loss under these conditions, due to the limited capacity of older adults to produce sweat[16,17]. Such an observation raises the need to identify alternative solutions that could be recommended to alleviate cardiovascular strain of older adults during heatwaves. It would be especially important to identify such interventions for people with cardiovascular disease, since this population is at an even greater risk of hospitalisation and mortality during periods of extreme heat. The aim of this study is to evaluate simple and ecological interventions to alleviate cardiovascular strain of adults with coronary artery disease.

2- Relevant literature review

The devastating health impacts of extreme heat events are clear across the globe. For example, a heatwave caused 70 000+ excess deaths in Europe during the summer of 2003. Mortality/morbidity rates during heatwaves are especially elevated in people with coronary artery disease (CAD)[18]. A lower sweating capacity that occurs with ageing renders CAD patients more vulnerable to

overheating, potentially predisposing them to ischemic events in extreme heat[14,19]. Risk may be further compounded in CAD patients prescribed cardio-selective beta-blockers, due to a blunted skin blood flow that may further compromise heat dissipation[20,21].

By far the most effective cooling strategy during a heatwave is air conditioning (AC) use[14]. However, access to AC is not universal and economical concerns limit its use by those who do have access[22]. Reliance upon AC also places significant burden on the electrical grid, which can cause brown-outs or black-outs during periods of extreme heat, and it adds a substantial thermal load to the environment [23,24]. In contrast, electric fans offer a cooling strategy with a 50-fold lower power requirement (55-100 W vs. 1500-5000 W) and cost ($64/year vs. $850-$2950/year) compared to AC. The energy benefits of this approach are two-fold: the individual is targeted instead of the surrounding space, and no active cooling of the air is required. During the 1990s, fan distribution programs targeted the most vulnerable in cities such as New York and Chicago as part of their heat wave management strategy[25]. But these programs were discontinued due to concerns that fans paradoxically increase the risk of heat-related illnesses. In fact, all major public health agencies (e.g. WHO, CDC) now insist that fans should be turned off when ambient temperature exceeds ~35°C[5-9] because they can supposedly speed the onset of heat exhaustion and critically exacerbate dehydration. It should be noted that, despite such recommendations, a 2012 Cochrane review concluded there is no evidence to support or refute the use of electric fans during heatwaves[26].

The notion that fan use may be detrimental in extremely hot conditions mostly likely arises from the understanding that as air temperature exceeds skin temperature (at ~35°C), more heat will flow into the body with additional air movement. After all, this is the basis upon which fan-assisted ovens accelerate cooking time. However, unlike a turkey in an oven, humans secrete sweat on to the skin surface when heated and the subsequent evaporative heat loss serves to greatly cool the body. Moreover, this evaporation is greatly enhanced with increased air velocity; leading to the evaporation of sweat that would otherwise sit on the skin, or drip off the body and provide no cooling. Therefore, it becomes clear that only when the additional heat gain with a fan cannot be counterbalanced by greater evaporative heat loss will fans accelerate body heating. Within this context, there are physiological limitations to sweating which will impact the efficacy of fan use. Age-related decrements in sweat output are progressively observed as early as 40 years of age [27], and while very little is known about sweating responses in individuals with coronary artery disease, the decreased ability to increase cardiac output sufficiently to maintain adequate levels of skin blood flow in CAD patients, particularly those prescribed cardio-selective beta-blockers, could further compromise heat dissipation.

An initial study in young healthy adults (20-30 years of age) was conducted to determine if fan use modifies the highest humidity that can be tolerated before increases in cardiovascular (i.e., heart rate) and thermal (i.e. core temperature) strain are observed. Compared to no fan use, the relative humidity (RH) at which cardiovascular strain occurred was ~20% RH higher with a fan at 36°C, and ~10% RH higher with a fan at 42°C (Fig. 1). Moreover, thermal strain was only observed at 36°C without a fan, and at a ~10% higher RH at 42°C with a fan (Fig. 1; right). These results demonstrate that fan use might be beneficial during heatwaves, even at high ambient temperatures of 42°C, since it delayed the point at which cardiovascular and thermal strain occurred. Since younger adults are generally not considered at risk during heat waves, we performed a follow-up study that assessed the efficacy of fan use for cooling healthy older (60-80 years) adults during a simulated heatwave at 42°C[2]. In contrast to the findings in younger adults, we found that fan use resulted in a higher heart rate and core temperature in older individuals (Fig. 2). In this case, fan use was detrimental, an observation that is likely explained by age-related reductions in sweating capacity[2,16,17].

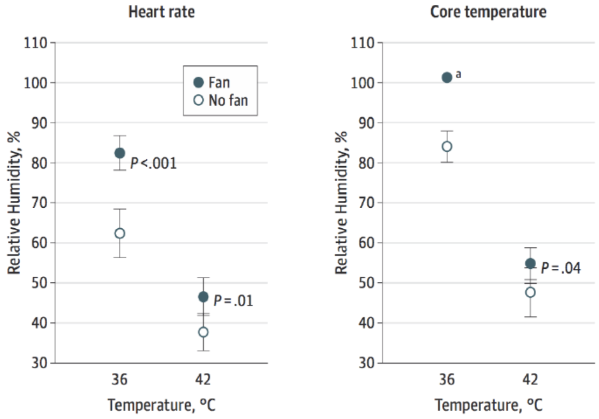

Fig 1. Relative humidity at which cardiovascular (heart rate, left) and thermal (core temperature, right) strain occur without (white circles) and with (filled circles) fan use in young adults, at 36°C and 42°C. P-values given for fan/no fan comparisons [3].

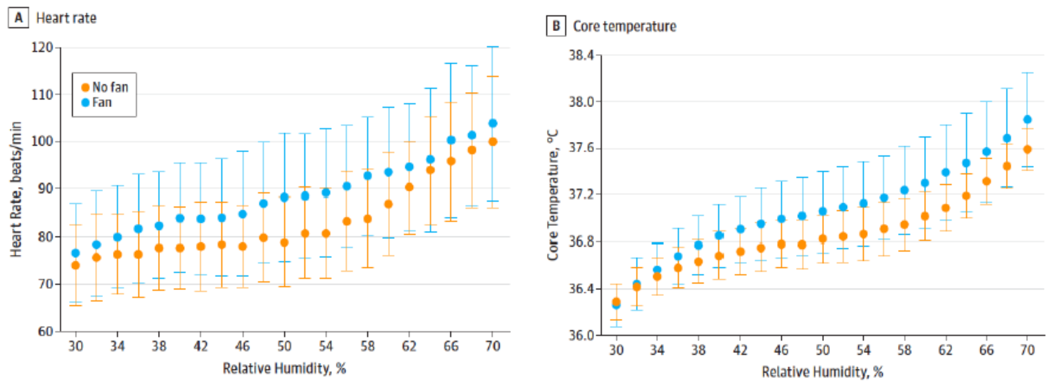

Fig 2. Heart rate (A) and core temperature (B) of older (60-80 y) adults exposed to an air temperature of 42°C with increasing relative humidity, with (blue) and without (orange) fan use [2].

Two key findings emerge from these studies; a) fan use has clear protective potential in young healthy people even at temperatures that far exceed the current public health guidelines (e.g. WHO); and b) the efficacy of fan use for cooling is dependent on the ability to physiologically wet the skin and as such fans become detrimental when used alone in older individuals because of impairments in sweating that occur with ageing[28,29]. It stands to reason, however, that age-related reductions in sweating can be compensated in older individuals by simply applying water to the skin surface. This would in turn greatly increase the cooling effect of fan use. Since heat loss capacity will likely be most limited in CAD patients prescribed beta-blockers secondary to a blunted skin blood flow, the benefits of skin wetting with fan use may be most pronounced in this group during heatwave conditions. However, the efficacy of skin wetting with or without fan use

to alleviate thermal and/or cardiovascular strain of CAD patients during heat waves has not been evaluated.

3- Objectives and evaluation criteria

3.1. Primary objective

The primary objective of this study is to identify the optimal cooling strategy to alleviate cardiovascular strain of CAD patients exposed to typical North American heatwave conditions (38°C with 60% RH). Cardiovascular strain will be quantified using rate pressure product, calculated as heart rate × systolic blood pressure. To address this objective, we will test the following hypothesis: During simulated heatwave conditions of 38°C and 60% RH, a control (no intervention) condition will result in a greater increase in rate pressure product compared to conditions of: fan use alone; skin wetting alone; fan use + skin wetting.

3.2. Secondary objective

A secondary objective is to identify the optimal cooling strategy to alleviate cardiovascular strain of CAD patients exposed to typical Australian heatwave conditions (46°C with 10% RH). Cardiovascular strain will again be quantified using rate pressure product. To address this objective, we will test the following hypothesis: During simulated heatwave conditions of 46°C and 10% RH, a control (no intervention) condition will result in a greater change in rate pressure product relative to a condition of skin wetting.

The effect of fan use (with or without skin wetting) will not be evaluated under Australian heatwave conditions. Preliminary data collected in young adults and provided by collaborator Prof. Ollie Jay (University of Sydney) demonstrate that fan use during these conditions results in a greater increase in rate pressure product compared to no fan use (Fig. 3).

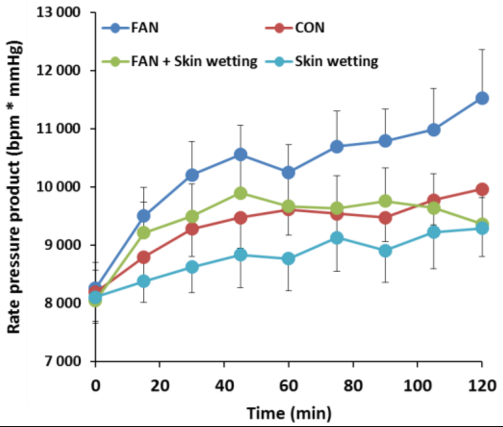

Fig 3. Rate pressure product (heart rate × systolic blood pressure) of young healthy adults (18-30 years) during a 2 hour exposure at 46°C and 10% RH. The data demonstrate that, under these conditions, fan use (FAN, dark blue) results in greater rate pressure product compared to: no intervention (CON, red); fan with additional skin wetting (FAN + skin wetting, green) and; skin wetting alone (light blue). Values are mean ± SE (n=12).

These observations can likely be explained by the fact that sweat evaporation is already maximal at such high heat and dry humidity conditions. As such, increased air velocity with fan use under these conditions greatly increases convective heat gain without resulting in greater sweat evaporation. Considering that older adults with CAD are likely to have a compromised sweating ability, we would not expect any benefit of fan use in this population during simulated heatwave conditions of 46°C and 10% RH. In fact, we would expect fan use to result in greater cardiovascular strain. For this reason, we will not evaluate the effect of fan use under these conditions.

3.3. Exploratory objectives

A tertiary objective is to examine if beta-blockers and alter the effectiveness of cooling interventions during each simulated heat wave conditions. To address this objective, we will compare a group of CAD patients prescribed beta-blockers to a group of CAD patients not taking beta-blockers. To account for the potential confounding influence of calcium channel blockers, patients prescribed calcium channel blockers will be asked to withhold this medication for 48 hours prior to each study visit.

Another tertiary objective is to evaluate how the interventions affect thermal and cardiovascular responses during each simulated heatwave condition.

3.4. Primary outcome

The primary endpoint will be the change in rate pressure product (heart rate x systolic blood pressure) from baseline (pre-exposure) to the end of a 3 hour simulated North American heatwave exposure (38°C and 60% RH).

3.5. Secondary outcome

The secondary endpoint will be the change in rate pressure product (heart rate x systolic blood pressure) from baseline (pre-exposure) to the end of a 3 hour simulated Australian heatwave exposure (46°C and 10% RH).

3.6. Exploratory outcomes

Tertiary endpoints will include, for each simulated heatwave condition, the change in rate pressure product from baseline (pre-exposure) to the end of the 3 hour simulated heatwave exposures between the group of CAD patients prescribed beta-blockers and the group of CAD patients not taking beta-blockers. Tertiary endpoints will also include: core and skin temperatures, heart rate, blood pressure, cardiac output, skin blood flow, local sweat rate, whole-body sweat rate, thermal comfort and thermal sensation.

**OBJECTIVES**	**EVALUATION CRITERIA**
Primary	
What is the optimal intervention to alleviate cardiovascular strain of CAD patients during North American heat wave conditions?	*Change in rate pressure product from baseline to the end of a 3 hour exposure at 38°C and 60% RH.*
Secondary	
What is the optimal intervention to alleviate cardiovascular strain of CAD patients during Australian heat wave conditions?	*Change in rate pressure product from baseline to the end of a 3 hour exposure at 46°C and 10% RH.*
Exploratory	
Do beta-blockers alter the effectiveness of cooling interventions during North American heat wave conditions in patients with CAD?	*Change in rate pressure product from baseline to the end of a 3 hour exposure at 38°C and 60% RH between a group of CAD patients prescribed beta-blockers vs. a group of CAD patients not taking beta-blockers.*

OBJECTIVES	EVALUATION CRITERIA
Do beta-blockers alter the effectiveness of cooling interventions during Australian heat wave conditions in patients with CAD?	*Change in rate pressure product from baseline to the end of a 3 hour exposure at 46°C and 10% RH between a group of CAD patients prescribed beta-blockers vs. a group of CAD patients not taking beta-blockers.*
How do the cooling interventions affect thermal and cardiovascular responses during simulated heatwave conditions?	*Change in the following variables during each of the heatwave exposures: core and skin temperatures, heart rate, blood pressure, cardiac output, skin blood flow, local sweat rate, whole-body sweat rate, thermal comfort and thermal sensation.*

5- Methods

5.1 Study design

Participants will be asked to volunteer for 1 preliminary visit, 1 fasting blood sample visit and 6 study visits. Participants will first undergo the 4 study visits for the simulated North American heatwave condition to minimize attrition for the primary objective. Participants will subsequently undergo the 2 study visits for the simulated Australian heatwave condition. Within each heatwave condition (North American, Australian) the sequence under which participants will undergo the study visits will be randomized. Prior to each visit, participants will be asked to refrain from strenuous physical activity, alcohol, and caffeine for 12 h. To ensure there is no carry-over effect, study visits will be performed on separate days, with at least 72 h between visits. Participants prescribed calcium channel blockers will be asked to withhold this medication for 48 hours prior to each study visit.

During the preliminary visit, participants will be invited to the laboratory and will be given the chance to familiarize themselves with all of the procedures and equipment involved in the study. They will also be given time to read the consent form and ask any questions they may have regarding their participation in the study. If they agree to participate, they will be asked to sign the consent form. Participants will then be asked to fill out a medical history questionnaire, following which we will measure their body weight and height, their resting heart rate and rhythm, and their resting blood pressure. A fasting blood sample will also be drawn.

During the study visits, participants will enter an environmental chamber maintained at either 38°C and 60% RH or 46°C and 10% RH. For each visit, the participant will remain within the environmental chamber and will rest in a seated position for 3 hours. The 6 study visits will consist of the following:

1) 38°C and 60% RH, no intervention (control)
2) 38°C and 60% RH, with fan use
3) 38°C and 60% RH, with skin wetting
4) 38°C and 60% RH, with fan use and skin wetting
5) 46°C and 10% RH, no intervention (control)
6) 46°C and 10% RH, with skin wetting

5.2 Description of the population

Participants will be male or female, aged between 50 and 80 years of age, with stable coronary artery disease.

5.2.1 Inclusion criteria
- History of angiographic coronary disease (≥70% arterial diameter narrowing of at least one major epicardial coronary artery) and/or prior coronary revascularization and/or documented prior acute coronary syndrome and/or stable angina and/or perfusion defect during exercise testing.
- No CAD-related hospitalisations or changes in cardiac medications or change in pattern of angina for at least 3 months prior to enrolment.

5.2.2 Exclusion criteria
- Body mass index ≥35 kg/m^2.
- Currently undertaking estrogen therapy.
- Evidence of current fluid and electrolyte disorders, anemia, abnormal thyroid function, arrhythmias, renal disease, liver disease, cerebrovascular disease, significant pulmonary disease, endocrine abnormalities, significant cognitive impairment, psychiatric disorder, substance abuse, degenerative neurological condition or any other medical condition deemed to pose risk during the proposed experiments.
- Type 2 diabetes not controlled by medication and/or severe neuropathy and/or severe retinopathy and/or insulin therapy.
- Uncontrolled hypertension (>180/110 mmHg).
- Recent (<3 months) coronary bypass surgery.
- Ejection fraction <40% and/or clinical evidence/history of heart failure.
- Significant valvular heart disease
- Resting ECG abnormalities interfering with observation of ST segment changes during testing.

5.2.3 Strategy for recruiting and retaining participants in the study
Potential participants will be recruited through the following strategies:
1) Approved advertisements. The advertisements will instruct interested individuals to contact, by phone or email, a member of the research team.
2) Phone calls to previous participants which gave their consent to be contacted for new research studies.
3) Phone calls to potential participants which gave their consent to be contacted about research studies during their annual medical visit.
4) Direct contact with patients enrolled in the ÉPIC prevention clinics. A member of the research staff will regularly attend the ÉPIC prevention clinics to provide information regarding the study. Interested participants will be given a copy of the approved advertisements and will be instructed to contact, by phone or email, a member of the research team

Once an interested individual contacts us, a member of the research staff will assess their eligibility to participate in the study by phone. If interested individuals meet the inclusion criteria, they will be invited to visit the laboratory for a preliminary visit. During this visit, a member of the research staff will familiarize interested individuals with all of the procedures and measurements of the study. Interested individuals will be given the opportunity to ask questions and they will also be given the time they need to read the consent form. If they decide to participate in the study, they will be asked to sign the consent form and fill out a medical history. Final confirmation of their eligibility to participate in the study will be determined by measuring their resting heart rate/rhythm and blood pressure and obtaining the results of a fasting blood sample.

5.3 Research protocol

5.3.1 Description of the visits

Upon awakening the day of a study visit, participants will be asked to swallow an ingestible telemetric pill which will have been provided to them beforehand. Upon arrival to the laboratory, participants will be asked to provide a urine sample, self-insert a rectal temperature probe and weigh themselves nude (in private). Participants will then be instrumented following which they will rest in the seated position for a 10 min baseline period at a room temperature of 24°C. Following this baseline period, they will enter an environmental chamber maintained at either 38°C and 60% RH or 46°C and 10% RH and will be seated on a mesh chair that will allow for convective airflow. Participants will remain seated for 3 hours within the environmental chamber while the relevant cooling intervention is administered. During the exposure, heart rate will be measured continuously from an ECG signal and blood pressure measurements will be taken every 20 minutes by automated auscultation of the brachial artery. Rate pressure product (**RPP**) will subsequently be calculated at baseline and every 10 minutes during the 3 hour exposure (see arrows in timeline below). Participants will be provided with 3 ml/kg of water every hour. At the end of the 3 hour period, participants will exit the environmental chamber to be de-instrumented and will be asked to weigh themselves nude in private to calculate whole-body sweat rate. During the study visits with fan use, an 18" diameter fan will face the participant from a distance of 1.0 m to provide an airflow of 4.0 m/s. During the study visits with skin wetting, tap water (~18°C) will be sprayed to the forehead, face, arms, lower legs and neck with a bottle every 5 minutes.

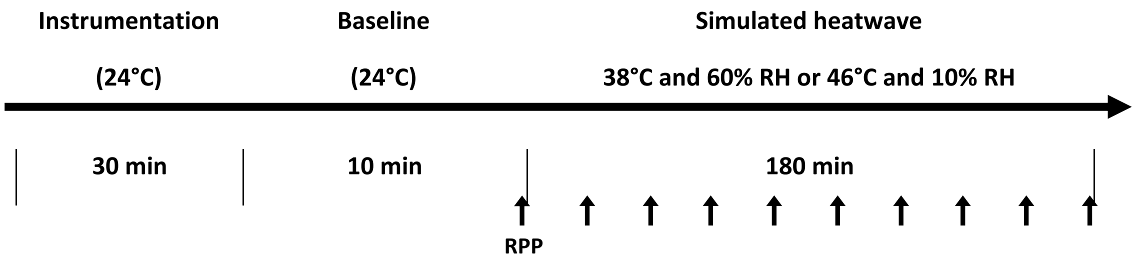

5.4 Measurements

1) Body weight will be measured during the preliminary visits, as well as before and at the end of each study visit with a scale.

2) Body height will be measured during the preliminary visit with a stadiometer.

3) Blood samples will be drawn from a sterile catheter inserted into a peripheral vein.

4) Heart rhythm will be acquired during the preliminary visits, as well as continuously during the study visits from an ECG signal. Heart rate will also be measured continuously during the study visits with a monitor placed around the chest.

5) Blood pressure will be measured during the preliminary visits, as well as every 10 minutes during the study visits by automated auscultation of the brachial artery.

6) Internal body temperature will be measured continuously during the study visits with an ingestible telemetric pill as well as with a rectal temperature probe.

7) Oral temperature will be measured by placing an oral thermometer under the tongue.

8) Skin temperatures will be measured continuously during the study visits by taping sensors to the skin surface.

9) Local sweat rate will be measured continuously during the study visits using plastic capsules attached to the skin surface and ventilated with dry air.

10) Skin blood flow will be measured continuously during the study visits on the forearm by laser-Doppler flowmetry.

11) Cardiac output will be measured every 20 minutes during the study visits with an inert gas rebreathing technique.

12) Urine specific gravity (as a measure of hydration status) will be determined from a urine sample collected at the beginning of each study visit.

13) Thermal comfort and sensation will be measured every 20 minutes during the study visits using visual scales.

**Measurements**	**Preliminary visit**	**Laboratory visits**
Information and consent	X	
Familiarization	X	
Medical history	X	
Body height	X	
Blood sample	X	X
Body weight	X	X
Heart rate and rhythm	X	X
Blood pressure	X	X
Internal body temperature (pill)		X
Internal body temperature (rectal)		X
Oral temperature		X
Skin temperatures		X
Local sweat rate		X
Skin blood flow		X
Cardiac output		X
Urine specific gravity		X
Thermal comfort and sensation		X

6- Statistics

6.1 Sample size

The sample size computation is based on comparison of the primary endpoint (change in rate pressure product from baseline to the end of a 3 hour simulated heatwave exposure to 38°C and

60% RH) between the 4 conditions of control, fan use alone, skin wetting alone, fan use + skin wetting.

The mean and standard deviation of the change in rate pressure product were taken from the preliminary data presented in Figure 3 and are expected to range between 1000 bpm x mmHg and 1700 bpm x mmHg for the mean and between 1700 bpm x mmHg and 2000 bpm x mmHg for the standard deviation. The standard deviation of the difference between conditions is expected to be similar.

The main interest is to compare the control condition with the 3 other conditions taken individually, which implies a significance level of 0.0167 (0.05 / 3). A difference of 1250 bpm x mmHg in change in rate pressure product between the control condition and any other condition is judged to be clinically relevant.

In this context a sample size of 30 participants will give 80% power to detect a difference of 1250 bpm x mmHg in change between the control condition and any other condition, assuming that the standard deviation of the difference between conditions is 2000 bpm x mmHg and using a paired t-test with a 0.0167 two-sided significant level. To account for an approximately 10% loss to follow-up rate, 34 participants will be included in the study. Of the 34 participants, 17 will be taking beta-blockers whereas the other 17 will not be taking beta-blockers.

6.2 Statistical analysis
Study variables will be presented using descriptive statistics. Mean, standard deviation, median, minimum and maximum will be presented for continuous variables. Number and percentage will be presented for categorical variables. Statistical assumptions underlying planned statistical tests will be examined and data transformation or non-parametric analysis might be used if appropriate. All statistical tests will be conducted at the 0.05 significance level unless specified otherwise.

Primary analysis: A one-way repeated analysis of variance (ANOVA) model will be used to compare the primary endpoint (change in rate pressure product under the 38°C and 60% RH heatwave exposure) between the conditions of: control, fan alone, skin wetting alone and fan + skin wetting. Contrasts under this model will allow for the three main comparisons:

- Control vs. fan alone
- Control vs. skin wetting alone
- Control vs. fan + skin wetting

These three comparisons will be tested at the 0.0167 significance level to control for the multiplicity of tests. This will be considered as the primary analysis of the study.

Since rate pressure product will be measured several times during the 3 hour simulated heatwave exposure, a two-way repeated measures ANOVA model on rate pressure product, having a term for condition, a term for time and a term for the condition x time interaction, might also be used to more precisely describe the pattern of change over the 3 hours of heat exposure across the conditions.

Secondary analysis: The change in rate pressure product from start to end of a 3 hour simulated heatwave exposure at 46°C and 10% RH will be compared between the control and skin wetting conditions using a paired t-test.

Exploratory analyses: To study the impact of beta-blockers, repeated measures ANOVA models on change in rate pressure product with a term for condition, a term for beta-blockers (yes/no) and a term for the interaction between condition and beta-blockers will be used for each heatwave condition. Other tertiary endpoints measured throughout the 3 hour exposures will be analyzed for each heatwave condition with a two-way repeated measures ANOVA model, having a term for condition, a term for time and a term for the condition x time interaction.

7- References

1. Meehl GA, Tebaldi C. More intense, more frequent, and longer lasting heat waves in the 21st century. *Science (New York, NY)* 2004;305:994-7.

2. Gagnon D, Romero SA, Cramer MN, Jay O, Crandall CG. Cardiac and Thermal Strain of Elderly Adults Exposed to Extreme Heat and Humidity With and Without Electric Fan Use. *JAMA* 2016;316:989-91.

3. Ravanelli NM, Hodder SG, Havenith G, Jay O. Heart rate and body temperature responses to extreme heat and humidity with and without electric fans. *JAMA* 2015;313:724-5.

4. Robine JM, Cheung SL, Le Roy S, Van Oyen H, Griffiths C, Michel JP, Herrmann FR. Death toll exceeded 70,000 in Europe during the summer of 2003. *C R Biol* 2008;331:171-8.

5. World Health Organization. Heat-Health Action Plans2008.

6. World Health Organization. Public health advice on preventing health effects of heat2011.

7. World Meteorological Organization, World Health Organization. Heatwaves and Health: Guidance on Warning-System Development2015.

8. United States Environmental Protection Agency. Excessive heat events guidebook2006.

9. Centers for Disease Control and Prevention. Extreme Heat Prevention Guide. 2006. (Accessed November 24, 2016, at https://www.cdc.gov/disasters/extremeheat/heat_guide.html, accessed November 24, 2016.)

10. Health Canada. Extreme heat events guidelines: Technical guide for health care workers. Ottawa, Ontario: Water Air and Climate Change Bureau, Healthy Environments and Consumer Safety Branch; 2011.

11. Institut national de prevention et d'éducation pour la santé (France). Canicule et fortes chaleurs : agir pour prévenir les risques. 2015. at http://inpes.santepubliquefrance.fr/10000/themes/evenement_climatique/canicule/canicule-agir.asp, accessed November 24, 2016.)

12. Centers for Disease Control and Prevention. Heat-Related Deaths - United States, 1999-2003. *Morbidity and Mortality Weekly Report (MMWR)* 2006;55:796-8.

13. Semenza JC, McCullough JE, Flanders WD, McGeehin MA, Lumpkin JR. Excess hospital admissions during the July 1995 heat wave in Chicago. *Am J Prev Med* 1999;16:269-77.

14. Semenza JC, Rubin CH, Falter KH, Selanikio JD, Flanders WD, Howe HL, Wilhelm JL. Heat-related deaths during the July 1995 heat wave in Chicago. *The New England journal of medicine* 1996;335:84-90.

15. Kenney WL, Craighead DH, Alexander LM. Heat waves, aging, and human cardiovascular health. *Med Sci Sports Exerc* 2014;46:1891-9.

16. Gagnon D, Crandall CG. Electric fan use during heat waves: Turn off for the elderly? *Temperature (Austin)* 2017;4:1-3.

17. Gagnon D, Romero SA, Cramer MN, Kouda K, Poh PY, Ngo H, Jay O, Crandall CG. Age Modulates Physiological Responses during Fan Use under Extreme Heat and Humidity. *Med Sci Sports Exerc* 2017;49:2333-42.

18. Hajat S, O'Connor M, Kosatsky T. Health effects of hot weather: from awareness of risk factors to effective health protection. *Lancet* 2010;375:856-63.

19. Kenny GP, Yardley J, Brown C, Sigal RJ, Jay O. Heat stress in older individuals and patients with common chronic diseases. *CMAJ* 2010;182:1053-60.

20. Gordon NF, van Rensburg JP, Russell HM, Kawalsky DL, Celliers CP, Cilliers JF, Myburgh DP. Effect of beta1 selective adrenoceptor blockade on physiological response to exercise. *Br Heart J* 1985;54:96-9.

21. Gordon NF, Myburgh DP, Schwellnus MP, van Rensburg JP. Effect of beta-blockade on exercise core temperature in coronary artery disease patients. *Med Sci Sports Exerc* 1987;19:591-6.

22. Sheridan SC. A survey of public perception and response to heat warnings across four North American cities: an evaluation of municipal effectiveness. *Int J Biometeorol* 2007;52:3-15.

23. Salamanca F, Georgescu M, Mahalov A, Moustaoui M, Wang M, Svoma BM. Assessing summertime urban air conditioning consumption in a semiarid environment. *Environ Res Lett* 2013;8:034022.

24. Salamanca F, Georgescu M, Mahalov A, Moustaoui M, Wang M. Anthropogenic heating of the urban environment due to air conditioning. *J Geophys Res Atmos* 2014;119:5949-65.

25. Bernard SM, McGeehin MA. Municipal heat wave response plans. *Am J Public Health* 2004;94:1520-2.

26. Gupta S, Carmichael C, Simpson C, Clarke MJ, Allen C, Gao Y, Chan EY, Murray V. Electric fans for reducing adverse health impacts in heatwaves. *Cochrane Database Syst Rev* 2012;7:CD009888.

27. Larose J, Boulay P, Sigal RJ, Wright HE, Kenny GP. Age-related decrements in heat dissipation during physical activity occur as early as the age of 40. *PLoS One* 2013;8:e83148.

28. Inoue Y, Nakao M, Araki T, Murakami H. Regional differences in the sweating responses of older and younger men. *J Appl Physiol (1985)* 1991;71:2453-9.

29. Inoue Y, Shibasaki M. Regional differences in age-related decrements of the cutaneous vascular and sweating responses to passive heating. *Eur J Appl Physiol Occup Physiol* 1996;74:78-84.

Summary of changes

1) USyd 2018-496: Use of fans and/or skin-wetting as a low cost cooling strategy for older adults during heatwaves

Date	Modifications
11/09/2018	- Edited total time for experimental study from 2hr to 3 hr<br>- Updated ambient conditions in hot dry heatwave to 45ºC, 15% RH<br>- Included measure of Urine specific gravity on arrival to laboratory to measure dehydration<br>- Updated total time for the study
15/10/2018	- Added blood collection at a pathology clinic if approved for study by study physician<br>Removed measure of body composition<br>- Modified the administration of water consumption, from 200 ml every 20 minutes to 3 ml per kg of body weight per hour<br>- Updated total remuneration for participants
19/11/2018	- Updated recruitment flyer
22/08/2019	-Added researchers: Amy Harwood, Connor Graham, Sarah Carter and Glenda Anderson to the study
01/11/2019	-Safety protocol updated to include HR termination criteria
14/09/2020	-Added researchers: Nicole Vargas, Timothy English, Lindsey Hunt
16/10/2020	-Updated safety protocol and heat safety protocol to specify instructions to call study Physician if specific ECG abnormalities are seen during trial
03/03/2021	-Update study information to reflect new address following laboratory relocation
19/05/2021	Updated recruitment flyers and social media advertisement
08/06/2021	Added new researchers: David Hutchinson and Angus Davis
16/06/2021	Added new recruitment flyer
01/12/2021	Updated all documents to include COVID safe procedures used in the laboratory
20/07/2023	Added new researchers: Grant Lynch

2) MHI 2019-2425: Identifying optimal cooling strategies for coronary artery disease patients during heatwaves

Date	Modifications
01/21/2019	- Added Georgia Chaseling as a collaborator<br>- Added the measurement of rectal temperature as an index of core temperature<br>- Modified the administration of water consumption, from 200 ml every 20 minutes to 3 ml per kg of body weight per hour
04/04/20219	- Removed diabetes as an exclusion criterion<br>- Added uncontrolled type 2 diabetes and/or severe neuropathy and/or severe retinopathy and/or insulin therapy as an exclusion criterion<br>- Changed the seat from a semi-recumbent chair to an an upright chair<br>- Increased the frequency of blood pressure measurements, from every 20 minutes to every 10 minutes
09/04/2019	- Modified the administration of skin wetting, from applying tap water (~18°C) every 10 minutes to the forehead, face, arms and neck using a cloth to spraying tap water (~18°C) to the forehead, face, arms, lower legs and neck with a bottle every 5 minutes.

Original statistical analysis plan

Montreal Health Innovations
Coordinating Center
A Division of the Montreal Heart Institute

STATISTICAL ANALYSIS PLAN

Protocol number: NA

USE OF FANS AND/OR SKIN WETTING AS A LOW COST COOLING STRATEGY FOR OLDER ADULTS WITH OR WITHOUT HEART DISEASE DURING HEATWAVES

Combination of studies #2018-496 (The University of Sydney) and 2019-2425 (Montreal Heart Institute)

Date of Final Statistical Analysis Plan: 19-JAN-2024

Montreal Health Innovations Coordinating Center (MHICC)
5000 Bélanger Street
Montréal, QC H1T 1C8
Phone: 514-461-1300 ext. 4043 / Fax: 514-461-1301

Signature Page

The Statistical Analysis Plan was prepared by:

Signature/Date: Malorie Chabot-Blanchet
Malorie Chabot-Blanchet (Jan 19, 2024 15:09 EST)

Malorie Chabot-Blanchet, M.Sc.
Biostatistician
MHICC

By signing below, I indicate that I have reviewed the Statistical Analysis Plan in its entirety and approve its contents.

Signature/Date: Daniel Gagnon
Daniel Gagnon (Jan 19, 2024 18:14 EST)

Daniel Gagnon, Ph.D.
Principal Investigator
Montreal Heart Institute

Signature/Date: Ollie Jay
Ollie Jay (Jan 22, 2024 10:33 GMT+11)

Ollie Jay, PhD
Professor of Heat and Health
The University of Sydney

Signature/Date: Marie-Claude Guertin
Marie-Claude Guertin (Jan 22, 2024 08:10 EST)

Marie-Claude Guertin, Ph.D.
Principal Biostatistician
MHICC

Revision History

Version	Date (DD-MMM-YYYY)	Author	Summary of Changes
Final	19-JAN-2024	Malorie Chabot-Blanchet	Initial version

TABLE OF CONTENTS

1 Introduction 5
2 Study Description 5
2.1 Study Design 5
2.2 Study Objectives 6
3 Datasets Analyzed 6
3.1 Full Analysis set 6
4 Efficacy Endpoints 6
4.1 Primary Efficacy Endpoint 6
4.2 Tertiary Efficacy Endpoints 7
5 Statistical Methodology 7
5.1 Statistical Considerations 7
5.2 Efficacy Analysis 7
5.2.1 Primary Analysis 7
5.2.2 Secondary Analysis 8
5.2.3 Tertiary Analysis 8
5.2.4 Other Subgroup Analysis 8
5.2.5 Other exploratory Analysis 9

LIST OF ABBREVIATIONS

**CAD**	Coronary Artery Disease
**REML**	Restricted Maximum Likelihood
**RH**	Relative Humidity
**SAP**	Statistical Analysis Plan

1 INTRODUCTION

The purpose of this statistical analysis plan (SAP) is to present the statistical methodology that will be used for the final analysis of the combined data from the 2018-496 and 2019-2425 studies performed respectively in Sydney, Australia and Montreal, Canada.

2 STUDY DESCRIPTION

2.1 Study Design

The study took place at two sites (Montreal and Sydney). Study participants were asked to volunteer for 1 preliminary visit, 1 screening visit and 4 to 8 study visits. During the study visits, participants were to enter an environmental chamber maintained at either 38°C and 60% relative humidity (RH) (typical North American heatwave condition) or 45°C and 15% RH (typical Australian heatwave condition). For each study visit, the participants were to remain within the environmental chamber and rest in a seated position for 3 hours under various conditions (cooling strategies). In Montreal, the 6 study visits were the following:

1) 38°C and 60% RH, no intervention (control)
2) 38°C and 60% RH, with fan use
3) 38°C and 60% RH, with skin wetting
4) 38°C and 60% RH, with fan use and skin wetting
5) 45°C and 15% RH, no intervention (control)
6) 45°C and 15% RH, with skin wetting.

In Sydney, participants were recruited for 2 preliminary visits and the 4 experimental visits under the North American heatwave exposure with the various cooling strategies. They were also provided with the option of doing additional visits that would be carried under the Australian heatwave exposure with the 4 cooling strategies, resulting in 2 additional visits compared to Montreal:

7) 45°C and 15% RH, with fan use
8) 45°C and 15% RH, with fan use and skin wetting.

In Montreal, participants were to first undergo the 4 study visits for the simulated North American heatwave exposure to minimize attrition for the primary objective. Participants were subsequently to undergo the study visits for the simulated Australian heatwave exposure. Within each heatwave exposure (North American, Australian) the sequence under which participants would undergo the study visits was randomized. In Sydney, participants were to undergo the 8 study visits in a randomized sequence. Prior to each visit, participants were asked to refrain from strenuous physical activity, alcohol, and caffeine for 12 h. To ensure there is no carry-over effect, study visits were to be performed on separate days, with at least 72 h between visits. Participants prescribed calcium channel blockers were asked to withhold this medication for 48 hours prior to each study visit.

In Montreal, male or female participants aged between 50 and 80 years of age with stable coronary artery disease were to be included in the study. In Sydney, male or female participants aged 60 years or older were to be included in the study.

2.2 Study Objectives

Primary objective :
The primary objective of this study is to identify the optimal cooling strategy to alleviate cardiovascular strain of participants exposed to typical North American heatwave conditions (38°C with 60% RH). Cardiovascular strain will be quantified using rate pressure product, calculated as heart rate × systolic blood pressure. To address this objective, we will test the following hypothesis: During simulated heatwave conditions of 38°C and 60% RH, a control (no intervention) condition will result in a greater increase in rate pressure product compared to conditions of: fan use alone; skin wetting alone; fan use + skin wetting.

Secondary objective:
A secondary objective is to identify the optimal cooling strategy to alleviate cardiovascular strain of subjects exposed to typical Australian heatwave conditions (45°C with 15% RH). Cardiovascular strain will again be quantified using rate pressure product.

Tertiary (exploratory) objectives:
A tertiary objective is to examine if beta-blockers alter the effectiveness of cooling interventions during each simulated heatwave conditions. To address this objective, we will compare a group of CAD patients prescribed beta-blockers to a group of CAD patients not taking beta-blockers. To account for the potential confounding influence of calcium channel blockers, patients prescribed calcium channel blockers were asked to withhold this medication for 48 hours prior to each study visit.

Another tertiary objective is to evaluate how the cooling strategies affect thermal (such as core temperature and whole-body sweat rate), and perceptual responses (such as thermal comfort and thermal sensation).

3 Datasets Analyzed

3.1 Full Analysis set

The Full Analysis set consists in all eligible subjects who underwent the control study visit and at least one other experimental visit within the same heatwave exposure (see section 2.1).

4 Efficacy Endpoints

4.1 Primary Efficacy Endpoint

The primary endpoint will be the change in rate pressure product (heart rate x systolic blood pressure) from baseline (pre-exposure) to the end of a 3-hour simulated heatwave exposure.

4.2 Tertiary Efficacy Endpoints

The tertiary endpoints will be the change from baseline to the end of a 3-hour simulated heatwave exposure in: core temperature, whole-body sweat rate (whole-body sweat loss/duration of heat exposure), thermal comfort and thermal sensation.

5 STATISTICAL METHODOLOGY

5.1 Statistical Considerations

Statistical analyses will be performed using SAS Version 9.4 or higher. Unless otherwise specified, all statistical tests will be two-sided and performed at a significance level of 0.05.

Prior to all parametric analyses, basic assumptions will be checked and if they are violated, non parametric analyses will be performed to confirm results from the parametric analyses. If outliers are identified, analysis with and without the outliers may be presented.

In the event that a participant did not complete the 180 minutes heatwave exposure, the change from baseline will be calculated using the last measure available.

5.2 Efficacy Analysis

All efficacy analysis will be conducted on the Full Analysis set.

5.2.1 Primary Analysis

The following descriptive statistics will be presented for the primary endpoint (change from baseline in rate pressure product) under the 38°C and 60% RH heatwave exposure: mean, standard deviation, median, Q1, Q3, minimum and maximum.

A restricted maximum likelihood (REML)-based linear mixed model including a term for condition and accounting for repeated measures, will be used to compare the change from baseline in rate pressure product under the 38°C and 60% RH heatwave exposure under the following conditions: control, fan alone, skin wetting alone and fan + skin wetting. Contrasts under this model will allow for the three main comparisons:

- Control vs. fan alone
- Control vs. skin wetting alone
- Control vs. fan + skin wetting

Differences between the control condition and each of the 3 other conditions will be tested and presented with point estimate along with 98.33% confidence intervals. These three comparisons will be tested at the 0.0167 significance level to control for the multiplicity of tests.

The assumption of normality underlying this model will be checked.

5.2.2 Secondary Analysis

Descriptive statistics (mean, standard deviation, median, Q1, Q3, minimum and maximum) will be presented for the change in rate pressure product from baseline to end of a 3-hour simulated heatwave exposure at 45°C and 15% RH. The change in rate pressure product will be analyzed using a REML-based linear mixed model including a term for condition and accounting for repeated measures. Contrasts under this model will allow for the three following comparisons:

- Control vs. fan alone
- Control vs. skin wetting alone
- Control vs. fan + skin wetting

The difference between the control condition and each of the 3 other conditions will be presented with point estimate along with 95% confidence intervals.

5.2.3 Tertiary Analysis

To study the impact of beta-blockers on the effect of the cooling interventions, change in rate pressure product will be analyzed using a REML-based linear mixed model, accounting for repeated measures, including a term for condition (control, fan alone, skin wetting alone and fan + skin wetting), a term for beta-blockers (yes/no) and a term for the interaction between condition and beta-blockers. This interaction will be tested at the 0.10 significance level and will determine whether the condition effect is impacted by taking beta-blockers. Condition effects will be estimated and presented with 95% confidence interval within subgroup of beta-blockers (yes/no). This analysis will be conducted on the subset of subjects with coronary artery disease (CAD) under the 38°C and 60% RH heatwave exposure. The same analysis will also be conducted in the subset of subjects with CAD under the 45°C and 15% RH heatwave exposure, with condition being restricted to control and skin wetting.

Under each of the heatwave exposures separately, change from baseline in core temperature, whole-body sweat rate, thermal comfort and thermal sensation will be presented and analyzed as the primary endpoint using a REML-based linear mixed model, but 95% confidence intervals will be presented with the point estimates and comparisons will be tested at the 0.05 significance level.

5.2.4 Other Subgroup Analysis

Subgroup analyses will be performed for the change in rate pressure product, looking at the homogeneity of the condition effect across the following factors: Sex (Female/ Male) and Site (Montreal/ Sydney).

The subgroup analyses will be conducted using a REML-based linear mixed model, accounting for repeated measures, including a term for condition, a term for the subgroup factor and the condition x subgroup factor interaction. This interaction will be tested at the 0.10 significance level and will determine whether the condition effect is impacted by the presence of the factor. Condition effects will be estimated and presented with 95% confidence interval within subgroups.

The subgroup analysis will be conducted separately under each of the heatwave exposures and will be considered exploratory.

5.2.5 Other exploratory Analysis

To take into account a potential acclimation effect that could occur during the summer, the change in rate pressure product will be analyzed using a REML-based linear mixed model, accounting for repeated measures, including a term for condition, a term for the season (Summer/Not Summer) and the condition x season interaction. This interaction will be tested at the 0.10 significance level and will determine whether the condition effect is impacted by the season. Condition effects will also be estimated and presented with 95% confidence interval within the season category.

The category "Summer" will be assigned to visits that occurred in June, July, August or September at the Montreal site and to visits that occurred in November, December, January, February, March or April at the Sydney site. Otherwise, the "Not Summer" category will be assigned. As season may vary across study visits of a same subject, it will be considered a within-subject factor in the analysis model.

This analysis will be conducted separately under each of the heatwave exposures and will be considered exploratory.
