# Supplementary material for: Thermal and Perceptual Responses of Older Adults With Fan Use in Heat Extremes: A Secondary Analysis of a Randomized Clinical Trial
Source: JAMA Netw Open. 2025 Jul 29;8(7):e2523810. doi: 10.1001/jamanetworkopen.2025.23810 (PMC12308431; doi:10.1001/jamanetworkopen.2025.23810)
Supplement: Supplement 3. — Data Sharing Statement [file jamanetwopen-e2523810-s003.pdf]

Data Sharing Statement

Data

Additional Information: ACTRN12618001913268 ACTRN12619000938101 NCT03832504
Data available: Yes
Data types: Deidentified participant data
How to access data: Raw data used for the analyses reported in this letter are freely available on request by contacting the corresponding authors: daniel.gagnon.3@umontreal.ca or ollie.jay@sydney.edu.au
When available: With publication

Supporting Documents

Document types: Other (please specify)
Additional Information: eMethods Protocol and statistical analysis plan
How to access documents: Supplements
When available: With publication

Additional Information

Who can access the data: Anyone requesting the data
Types of analyses: For any purpose
Mechanisms of data availability: Without investigator support
Any additional restrictions: None
